# Machine learning and game theory for cyber governance: Enhancing public opinion and regional sustainable development

**DOI:** 10.1371/journal.pone.0308317

**Published:** 2024-12-05

**Authors:** Podong Song, Wiseong Jin, Shaowei Chen, Xufang Hu, Kwisik Min, Shengchao Li, Senmong Li

**Affiliations:** 1 Kunming University, Kunming City, China; 2 Macao Polytechnic University, Macao, China; 3 Development and Planing Department of North Sichuan Medical College, Nanchong City, China; 4 Hanyang University, Seoul, South Korea; 5 School of Education Science, Zhaotong University, Zhaotong City, China; 6 Yichuan County Water Resources Bureau, Luoyang City, China; 7 Department of Chinese Language and Literature, Jeonbuk National University, Jeonju-si, Jeollabuk-do, Republic of Korea; 8 Gachon University, Seongnam, South Korea; Zhejiang Gongshang University, CHINA

## Abstract

Cyberspace is emerging as a critical living environment, significantly influencing sustainable human development. Internet public opinion is a crucial aspect of cyberspace governance, serving as the most important form of expressing popular will. However, perceiving public opinion can be challenging due to its complex and elusive nature. In this paper, we propose a novel framework for perceiving popular will, managing public opinion, and influencing people’s behavior, based on machine learning and game theory approaches. Our framework leverages deep learning techniques to analyze public opinion, active learning methods to reduce costs, and game theory to make optimal management decisions. We verify the effectiveness of our framework using empirical data collected from Chinese provinces Y and G, and provide theoretical support by analyzing the interrelationship between public opinion, online public opinion, and people’s behavior. Our framework can be applied inexpensively to studies in other regions, thereby offering valuable insights into cyberspace governance and public opinion management.

## 1. Introduction

By 2021, with more than half of the world’s population accessing the Internet through mobile devices, the governance of cyberspace has become a critical global concern [1]. According to the 51st Statistical Report on China’s Internet Development by the China Network Information Center (CNNIC), the number of Internet users in China has reached 1.051 billion, with an Internet penetration rate of 74.4%. On average, users spend 27 hours per week online. Weibo, China’s largest online social media platform, will have 586 million active users by the end of 2022, making it a prominent platform for expressing views and opinions [2]. The increasingly severe global cybersecurity situation and the frequent occurrence of information content security incidents make cyberspace, especially social media platforms, a fertile ground for the rapid spread and amplification of cyber public opinion due to its suddenness, destructive potential, and widespread impact. As a result, effective management of cyber public opinion has become a critical aspect of cyberspace governance.

Mismanagement of public opinion on the Internet can lead to misinformation, which can have a negative impact on people’s values and offline behavior. China’s major media reportedly debunked an average of 67 rumors per day [3]. This problem was particularly exacerbated during the COVID-19 epidemic when mismanagement of public opinion hampered effective prevention and control efforts. A notable example is the "Shuanghuanglian" incident, which caused panic and inappropriate offline behavior [4]. Conversely, if properly managed, online public opinion can play an important role in resolving social conflicts. By steering public opinion in the right direction, authorities can actively address concerns, dispel doubts, and engage with netizens to create a new platform for meaningful communication [5, 6]. This, in turn, strengthens government credibility, reduces social pressure, stabilizes order, and facilitates evidence-based policy making.

The formation of public opinion often begins with the awakening of citizens’ subjective consciousness, which can then evolve into popular will and finally solidify as public opinion, especially under the influence of significant events [7]. It is of paramount importance for governments to accurately perceive and effectively manage public opinion as it spreads and diffuses. However, this task is challenging due to the multifaceted nature of public opinion and the complexity of predicting its evolution. Traditional methods of gauging public opinion are both time-consuming and costly [8], requiring extensive surveys and data analysis, resulting in delayed updates and reduced accuracy. As a result, capturing public opinion in a timely and accurate manner remains a challenging endeavor. However, sentiment analysis has its difficulties because it requires the analysis of numerous texts, making it difficult for managers to determine the content and direction of opinion management.

Machine learning technology is developing rapidly, and its application has become a focus of scientific research, providing new methods and tools for various studies. One such application is to propose a deep neural network (DNN)-based architecture for online visualization and tracking of automated guided vehicles (AGVs) in the Internet of Things (IoT). This approach aims to strengthen cybersecurity in AGV state tracking, thereby improving decision-making capabilities and industrial productivity [9]. In addition, machine learning is used for fault detection and correction in induction motors, ensuring optimal motor performance and extending their lifetime [10]. The choice of loss function also plays a critical role in determining model effectiveness [11]. In the field of opinion analysis, machine learning has attracted increasing attention, especially as the Internet serves as an important platform for political discussions [12]. Numerous studies focus on monitoring and managing public opinion [13–16], using natural language processing techniques and social network analysis to study public sentiment on issues such as food safety [17]. During the COVID-19 epidemic, researchers used machine learning techniques to analyze public opinion in real time [18]. Sentiment analysis, facilitated by machine learning, is also instrumental in understanding public opinion [19]. For example, deep learning is used to perform sentiment analysis on COVID-19-related online discussions [20]. In addition, some scholars have applied aspect-based sentiment analysis to online reviews to help operators make effective decisions [21]. Game theory has gained prominence in public opinion management. Scientists have used evolutionary game theory to study public opinion on the doctor-patient relationship [14].

However, current research on public opinion analysis mainly focuses on analyzing the public opinion of specific events or specific groups [16], and few studies have analyzed public opinion across different regions. Due to the difficulty of obtaining public opinion, there are also limited studies that directly target public opinion. Previous studies have mostly focused on analyzing or managing public opinion [22], and have not explored the relationship between popular will and public opinion or the relationship between public opinion and people’s behavior, nor have they proposed a comprehensive framework to address these issues. Furthermore, the high cost of deep learning for sentiment analysis has been a challenge. Deep learning often requires a large amount of data for training [23], with some models even requiring hundreds of thousands of data points for training [24]. Additionally, deep learning, as supervised learning, requires labeling of training data, which is mainly done manually and can be time-consuming and costly. These challenges can lead to delays in accurately perceiving management priorities and affect decision-making processes.

**Fig 1:**
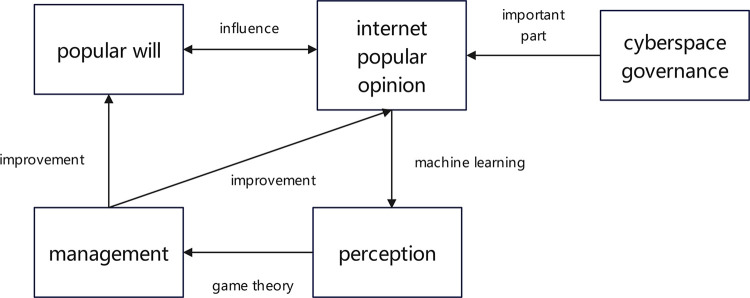
The framework for perceiving and managing popular will and public opinion.

This paper presents a novel framework (Fig 1) for sensing and managing public will and public opinion. The framework analyzes the interplay between public opinion, public will, and people’s behavior to identify key management components. Game theory models are used to analyze scenarios and propose effective solutions.

This paper makes several noteworthy contributions.

The paper puts forth a comprehensive "perception-management" framework for the analysis of public opinion and perception, which can be readily adapted to analogous research domains.

The analysis of public opinion in two representative Chinese regions provides a valuable and innovative comparative study. The data from these regions is processed using machine learning algorithms, which extract themes and sentiments with an impressive accuracy of 0.925, outperforming similar studies. The application of active learning reduces the cost of opinion analysis by 20%, resulting in a final model accuracy of 0.911. This significant cost reduction enhances the practicality and efficiency of the proposed analysis framework.

A systematic analysis of public opinion, public will, and people’s behavior is conducted to elucidate the relationships among these components and to identify areas that require management. Furthermore, the paper uses a game model to analyze the relevant management issues and proposes improvements to the current challenges in cyberspace governance based on the theoretical framework.

## 2. Related work

### 2.1. Machine learning

Latent Dirichlet Allocation (LDA) is a widely used technique in various fields. For example, it has been used to analyze factors influencing consumer satisfaction in the e-commerce of fresh produce [25], as well as media reports on COVID-19 vaccination in Korea [26]. In the area of opinion research, LDA has been applied in several studies, including investigations of popular food products on the Internet [27], comments on the social networking site Weibo [28], and trending topics related to COVID-19 based on knowledge graphs [29]. In addition, LDA has been used for the topic analysis of digital documents to improve the understanding of such materials [30]. In the context of climate change research, scholars have used LDA techniques to perform spatiotemporal difference analysis, which led to the identification of four prominent categories of climate change-related issues, with the public showing a notable concern for green development [31]. In addition, LDA has been instrumental in exploring the development paradox problem of world university rankings, analyzing it from four main perspectives: positional cognition, dialectical cognition, interest cognition, and cultural cognition [32]. LDA has proven valuable in analyzing the impact of bugs on users in software development, enabling software developers to gain a better understanding of user needs [33].

Deep learning techniques have gained widespread popularity and found applications in various fields. For example, researchers have used deep learning algorithms to achieve reliable fault recognition in induction motors, enabling trustworthy and effective decision-making methods [10]. Some scholars have used natural language processing techniques to analyze relevant research activities in the fields of computer science and information technology during the COVID-19 epidemic, and have achieved good performance [34]. In addition, deep learning has been instrumental in improving the resistance of automated guided vehicles to cyber-attacks, thereby enhancing decision-making capabilities and industrial productivity [9]. In the field of sentiment analysis, various studies have used different methods such as LSTM [20] and CNN [35] to analyze online discussions, proving these models to be valuable tools. Sentiment analysis has also been used to study the evolution of consumer purchase intentions [36]. Some scholars have used deep learning algorithms, including CNN and LSTM, to build intelligent systems to control the behavior of wastewater treatment systems through anomaly detection to support its decision-making, and achieve good performance [37]. In addition to deep learning, traditional machine learning algorithms have also proven effective, especially when combined with neural networks [38]. For example, machine learning methods were used to analyze opinion data from Twitter during the COVID-19 pandemic [18]. The authors compared and analyzed the performance of logistic regression, random forest, polynomial naive Bayes, and long- and short-term memory models. In addition, machine learning was applied to sentiment analysis for disease outbreak prediction, resulting in the development of improved prediction systems and health care coverage [39]. In addition, machine learning-based sentiment analysis accurately predicted trends in financial markets during the COVID-19 epidemic [40]. In particular, machine learning sentiment analysis during the COVID-19 pandemic can provide valuable public opinion guidance to government agencies and healthcare workers in their fight against COVID-19 [41].

This paper employs a combination of CNN and LSTM to extract themes and sentiment from public opinion data. Active learning techniques are also utilized to reduce the cost of supervised learning. Prior research has shown that active learning is an effective approach to reducing annotation costs and improving model accuracy. For instance, [42] conducted an empirical and theoretical analysis of active learning, while [43] proposed a new active learning strategy for deep neural networks that reduces the number of data annotations queried during training.

### 2.2. Game theory

Game theory is a widely used tool in various fields of research for modeling and analyzing decision scenarios. One of its most notable offshoots is evolutionary game theory, which emerged in the 1990s and integrates traditional game theory with Darwin’s theory of biological evolution. Unlike traditional game theory, evolutionary game theory does not assume complete rationality or information, but considers organisms as participants with limited rationality. These participants continuously learn, adjust their strategies, and engage in repeated games to maximize their interests [44]. In addition, evolutionary game theory emphasizes dynamic equilibrium [45], making it particularly well suited to studying complex system interactions and the evolution of individual strategies [46].

In practical applications, evolutionary games have been used to analyze the impact of government policies on promoting carbon capture and utilization. By examining the evolutionary patterns of stakeholders, meaningful insights are provided to guide government decision-making [47]. In addition, evolutionary game theory has proven beneficial in studying the purchasing strategy of bike-sharing, providing strategic support for improving profitability in the bike-sharing market [48]. In the field of public opinion research, scholars have also used game theory to address public opinion issues. For example, [14] applied game theory to analyze the dynamics of public opinion between doctors and patients, while [49] studied green buildings from the perspective of consumer preferences. In addition, [50, 51] developed game models to analyze public opinion problems. By applying game theory, researchers can gain valuable insights into the strategic behavior of different actors and design effective public opinion management programs.

### 2.3. Literature gaps

Prior research has employed LDA for the extraction of topics from social media text data, with the objective of investigating topics such as public opinion and university rankings during the course of the global pandemic caused by the SARS-CoV-2 virus. Nevertheless, there is a dearth of comparative analysis involving text data from two Chinese provinces simultaneously. Although previous studies have employed deep learning for sentiment analysis, few have utilized a simultaneous fusion of convolutional neural networks (CNN) and long short-term memory (LSTM) units with active learning strategies. While machine learning algorithms have been employed to analyze phenomena, few have endeavored to elucidate the underlying causes of problems. Moreover, there is a dearth of studies that propose solutions to identified problems based on game theory principles. A comprehensive literature review revealed several shortcomings in previous studies. Firstly, although numerous studies have employed machine learning algorithms to examine public opinion, few have addressed the governance of cyberspace. These studies only analyzed public opinion about a specific event, failing to provide an analysis from the perspective of public opinion and opinion management. Furthermore, previous studies have not prioritized enhancing the precision of the machine learning algorithms utilized for sentiment analysis, and their accuracy rates have not reached an optimal level. Additionally, no prior studies have put forth a "perception management" framework or analyzed data from two regions. In conclusion, although some studies have employed game theory to examine the conduct of online participants, few have utilized it for the analysis of public opinion and opinion management.

## 3. Theoretical foundations

### 3.1. Latent Dirichlet allocation (LDA)

**Fig 2:**
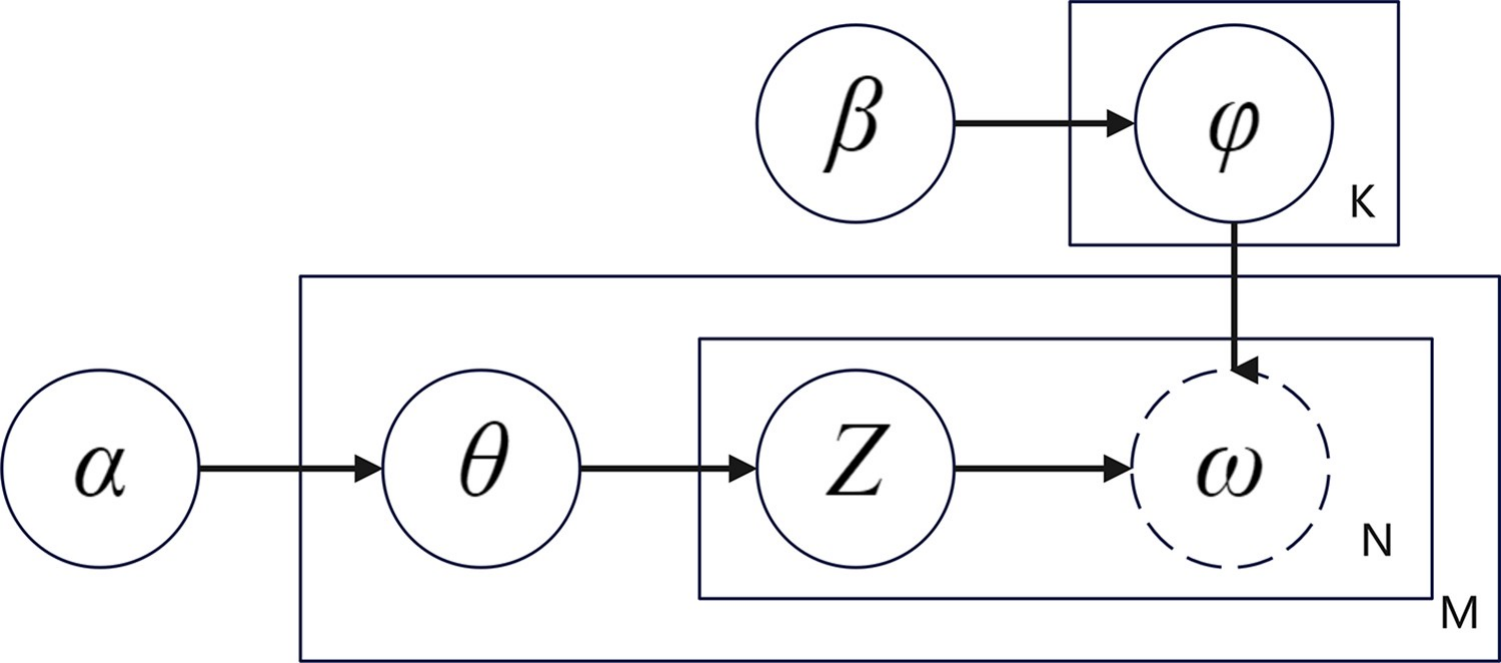
LDA model.

Latent Dirichlet allocation (LDA) is a document topic generation model [52] that is widely used in the field of semantic mining because of its ability to reduce the representation dimensionality of text [53]. The process is shown in Fig 2. First, a document d is selected, which consists of multiple words, each corresponding to a potential topic. The distribution of the probability of "text-topic", θ, is first generated with the parameter α satisfying the Dirichlet distribution; then the distribution of the probability of the topic corresponding to the words in document d, Z is generated from the polynomial distribution θ; the distribution of "topic-word" φ is generated with the β parameter satisfying the the Dirichlet distribution; finally, the probability of word ω is generated from the combined result of Z and φ. The rectangles in the figure represent repeated sampling, and M, N, and K all represent the number of times. In particular, the number of topics K needs to be given manually.

The LDA algorithm is an unsupervised model in which the number of topics is one of the most important input parameters and needs to be given manually. There are many ways to select the optimal number of topics, and this paper uses a perplexity to obtain the optimal number of topics, with the following formula.

$$perplexity(D)=exp\left\{-\frac{\sum_{d=1}^{M}log\;p(w_{d})}{\sum_{d=1}^{M}N_{d}}\right\}$$ (1)

where w_d_ denotes the word, p(w_d_) denotes the probability of the word in the document, N_d_ denotes the number of words, M denotes the number of documents and D denotes the set of all words in the document.

The higher the number of topics, the lower the value of perplexity will be, but it is not the case that a higher number of topics is better, otherwise, it will overfit. The optimal number needs to be chosen in combination.

### 3.2. Convolution Neural Networks (CNNs)

Convolution Neural Networks (CNNs) is a variation on ANNs [54] that use convolution to extract features. CNNs have been used with good results since they began to be used [55] and are currently the more popular neural network [56].

**Fig 3:**
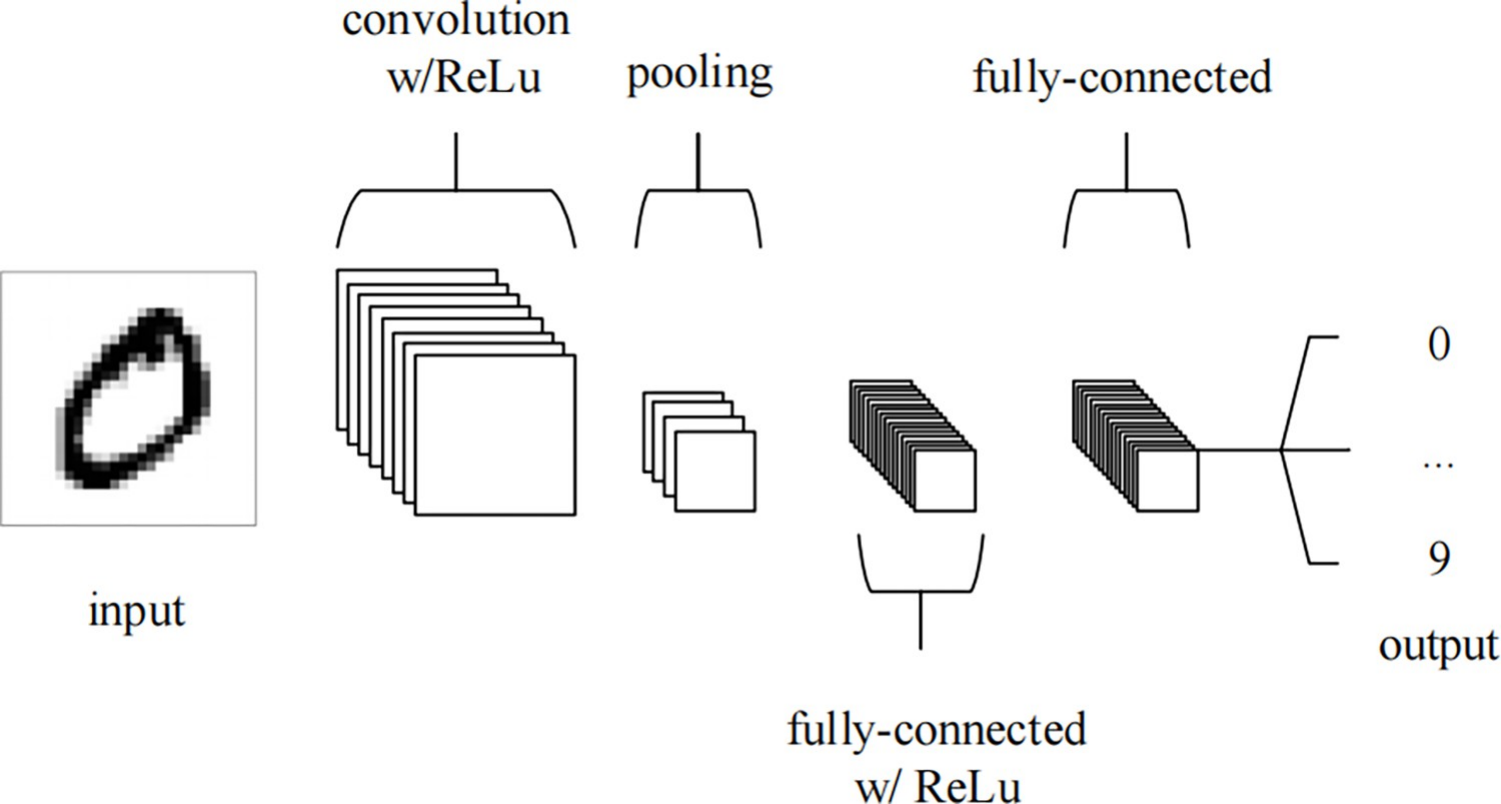
An simple CNN architecture.

The CNN is divided into three main layers: the convolutional layer—the main role is to extract features; the max pooling layer—the main role is to downsample without damaging the recognition results, and the fully connected layer—the main role is to classify. This is shown in the Fig 3 [54].

### 3.3. LSTM (Long Short Term Memory)

**Fig 4:**
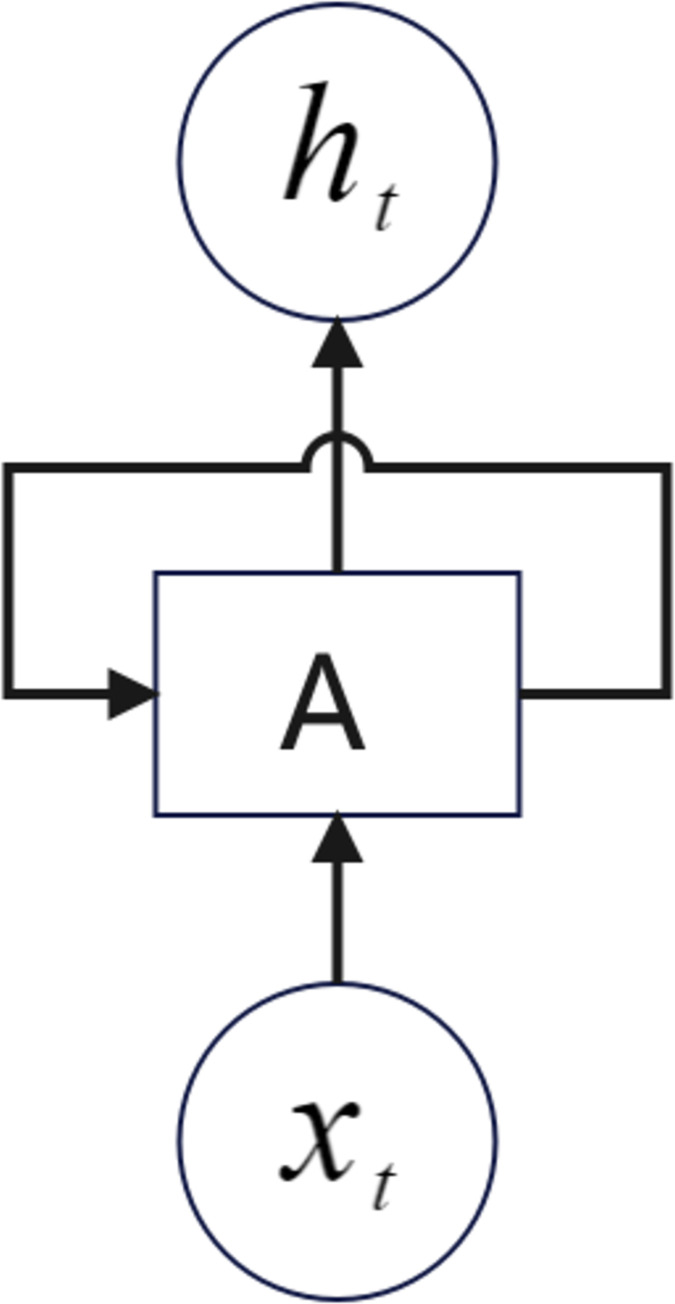
RNN model. where x_t_ is the input information at moment t and h_t_ is the input information at moment t. Neuron A can recursively call itself and pass the information at moment t−1 to moment t.

LSTM (Long Short Term Memory), an optimized form of RNN, was proposed [57] and improved [58] to excel in working with sequential data, especially when there is a connection between the preceding and following data. the RNN structure is shown in Fig 4 [59].

### 3.4. Active learning

Active learning (sometimes referred to as “query learning” in the statistical literature) is a subfield of machine learning. More broadly, it is an area of artificial intelligence. The key assumption is that if the learning algorithm can choose the data that is more interesting to it, rather than accepting all the data, then the algorithm will get the same effect as using all the training data with less training data?

The necessity of this property and algorithm is evident when one considers the fact that, in order for supervised learning to perform well, a substantial amount of training data is required to develop the model. In the current era of big data, it is not uncommon for a deep learning model to require 100,000 or more training data points. In some cases, obtaining this data can be challenging.

**Fig 5:**
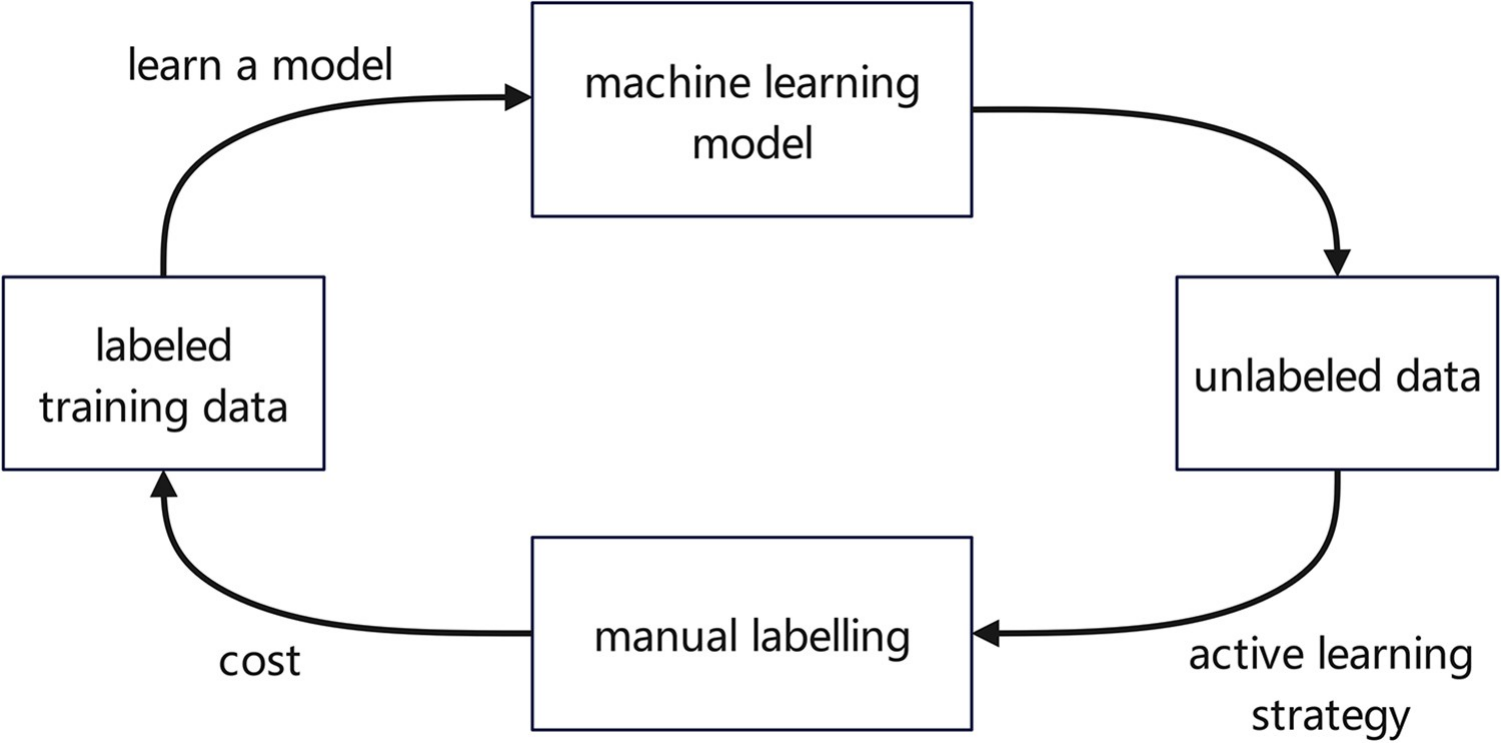
Active learning framework.

However, for a large part of the supervised learning tasks, the training data is very small, and it is also very expensive, and there are many training data that contain many errors. These will lead to difficulties in supervising learning, and it will not achieve the desired results. At this time, active learning is very helpful. What is shown in the Fig 5 is a basic model of active learning. Usually, active learning first uses a small amount of labeled data to input into the supervised learning model, and then the supervised learning model will use this part of the data as training data, and the trained model is not good enough. Next, you need to select data from Unlabeled data to enter the labeled data. The ultimate goal is to get the same results with less training data.

### 3.5. Performance evaluation

For the model of sentiment analysis, we use the [18, 60] approach for measurement, using a confusion matrix, to calculate the F1 score. For the effect of active learning, we measure the model effect of active learning by comparing the accuracy and F1 scores. In general, the accuracy and F1 scores decrease after using active learning, but if the decrease is in an acceptable range, it shows that active learning is helpful.

Classifier accuracy is measured with the help of a confusion matrix. This matrix provides the number of right and wrong predictions by comparing actual target values. It consists of four parameters: (1) true positive (TP): shows how many actual true values the model predicted as true; (2) true negative (TN): shows how many actual false values the model predicted as false; (3) false positive (FP): shows how many actual false values are predicted as true; and (4) false negative (FN): shows how many actual true values are predicted as false.


$$Accuracy=\frac{TP+TN}{TP+TN+FP+FN}$$ (2)

$$Precision=\frac{TP}{TP+FP}$$ (3)

$$Recall=\frac{TP}{TP+FN}$$ (4)

$$F1\;score=2*\frac{Precision*Recall}{Precision+Recall}$$ (5)

## 4. The framework for perceiving and managing popular will and public opinion

This study introduces an integrated framework, shown in Fig 1, designed for the perception and management of public opinion and popular will. This framework serves as the research methodology for this study and is organized into three core components: public opinion perception, analysis of the interrelationship between online public opinion, popular will, and human behavior, and formulation of management content models using game theory principles. The study draws on data from two Chinese provinces, Y and G. Province Y has abundant natural tourism resources but lags in economic development, while Province G has fewer natural resources but a higher level of economic prosperity. The selection of these provinces provides representative insights into balanced regional and sustainable development. To the best of our knowledge, this paper presents the first integrated "perception management" framework for public opinion and public will, offering a comparative analysis of online public opinion data from the representative provinces, Y and G. Furthermore, the proposed framework is generalizable and applicable to similar regions in other countries for public opinion research. In addition, this study employs active learning to demonstrate cost-effective measures in perceiving public opinion and public will, significantly reducing monetary and time expenditures. In summary, the contribution of the study is remarkable. The framework presented in Fig 1 encompasses several aspects. First, it underscores the importance of public opinion in cyberspace governance by illustrating the mutual influence of public opinion and public will. To address the challenge of unperceived public opinion, this paper uses machine learning technology to perform topic and sentiment analysis on extensive public opinion data, enabling timely and accurate perception. Based on the results of this analysis, the relationship between public opinion, public will, and offline behavior is explored to derive goals and directions for effective public opinion management. In addition, the study examines the behaviors and motivations of various stakeholders involved in public opinion and public will management. By introducing a game model, the paper proposes a method to improve both public opinion and the public will, thus extending the " perception management" framework. Detailed explanations of the machine learning model, the relationship diagram of public opinion, public will, and offline behavior, and the game model are provided in subsequent chapters. The first segment, public opinion perception, emphasizes the importance of data collection and analysis in understanding public opinion. Specifically, this study involves the extraction of text data from Weibo to gain accurate insights into public opinion. Then, in this study, the LDA algorithm is used to perform thematic analysis on the textual data to extract prevalent themes in public opinion. A combined CNN+LSTM model is then used for sentiment analysis of the text. The results of both theme extraction and sentiment analysis are combined to understand public opinion. In the next section, a complex examination of the multifaceted relationship between online public opinion, popular will, and human behavior is conducted. This analysis aims to identify the key factors influencing the management of public opinion by examining the correlation between public opinion and the online and offline behavior of individuals. Finally, the third segment of the framework concerns the application of game theory modeling. The first part of the framework focuses on the understanding of public opinion, while the second part delves into the analysis of public opinion management, aiming to identify the essential factors associated with its management. Consequently, the third section conducts a game-theoretic modeling analysis that provides an effective scheme and methodology for the efficient management of public opinion. To validate the proposed framework, this study uses public opinion data from provinces Y and G.

### 4.1. Public opinion perception

#### 4.1.1. Analysis framework for internet public opinion

**Fig 6:**
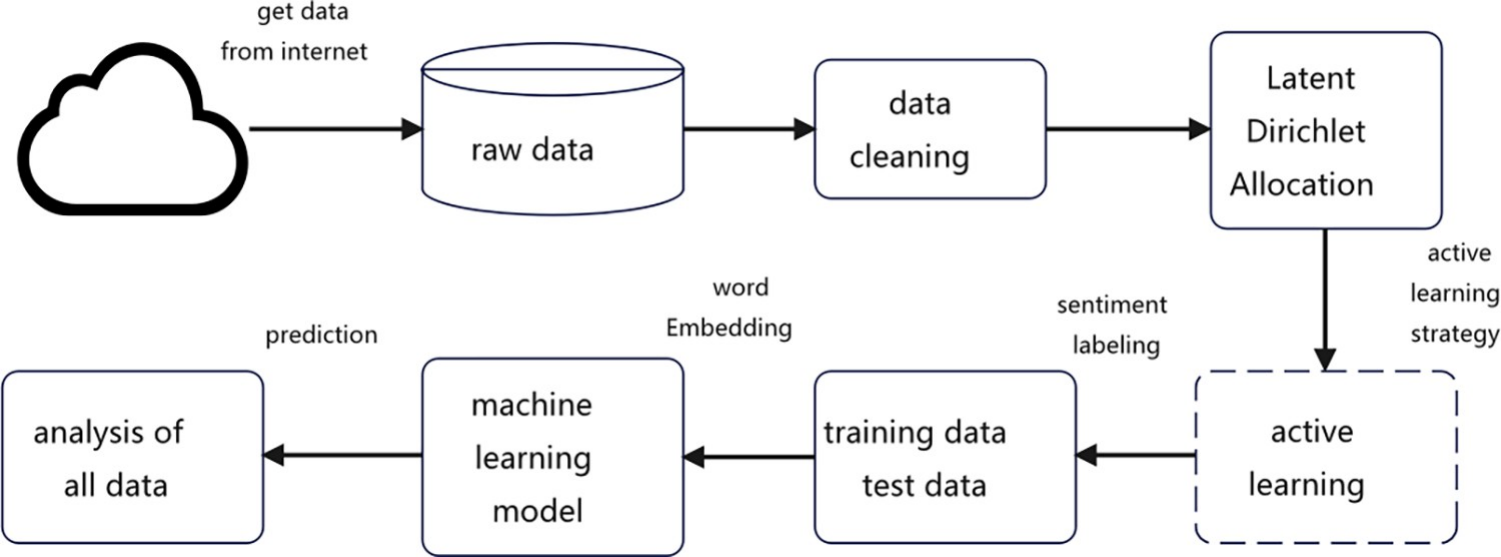
The analysis framework for internet public opinion.

Fig 6 presents the analysis framework for internet public opinion, which is composed of four primary stages: (1) data acquisition and pre-processing, (2) LDA topic analysis, (3) training of machine learning sentiment prediction models, and (4) data analysis.

When dealing with noisy data, it is critical to select an appropriate method that fits the specific problem and data characteristics. In addition, repeated experiments and evaluations are essential to ensure the validity and reliability of the processed data for machine learning tasks. The first step is to acquire relevant data from the Internet, followed by data cleaning and preprocessing. The data preprocessing phase includes noisy data processing, text segmentation, and word stemming. Dealing with noisy data is of paramount importance in machine learning and data analysis, as noise can adversely affect model performance. In this study, text comments from online social platforms are analyzed, and the processing of noisy data includes data cleaning, filtering irrelevant characters, removing stop words, and handling outliers. Given the noisy nature of text data in online comments, the data cleaning process removes duplicate data and handles missing values. Since comment content often includes emoticons, URLs, punctuation, tags, and @ symbols, it is imperative to filter out irrelevant characters before segmentation. To achieve this, regular expressions are used to extract the relevant text content from the original data set. Text segmentation is an important preliminary step before applying LDA topic modeling. In this study, we use the Jieba [61] segmentation tool to process the text data and derive the segmented data. To reduce the interference of irrelevant words, stemming processing is applied to the segmented data. Specifically, words that have no practical meaning but occur frequently in Chinese text, such as connectives and tone words, are identified as deactivated words and then removed. This ensures that meaningless words do not affect the model’s analysis results. In addition, special expression habits in Chinese text are addressed through manual sampling and verification. Using specialized domain knowledge, data filtering is performed to effectively eliminate the influence of noisy data. The resulting clean data is then used to generate word clouds and semantic relationship graphs, providing a deeper understanding of the data.

This paper employs a preprocessed dataset for topic extraction utilizing the Latent Dirichlet Allocation (LDA) model, an unsupervised learning model that necessitates a predefined number of topics. The perplexity value is employed as a reference point for determining the optimal number of topics. Prior to applying the LDA model, the dataset undergoes a preprocessing phase, during which irrelevant characters are filtered out, the text is split into individual words using the Jieba word segmentation tool, and stop words are removed. Subsequently, a word cloud and a semantic relationship graph are generated to visualize the meaningful words within the dataset.

**Fig 7:**
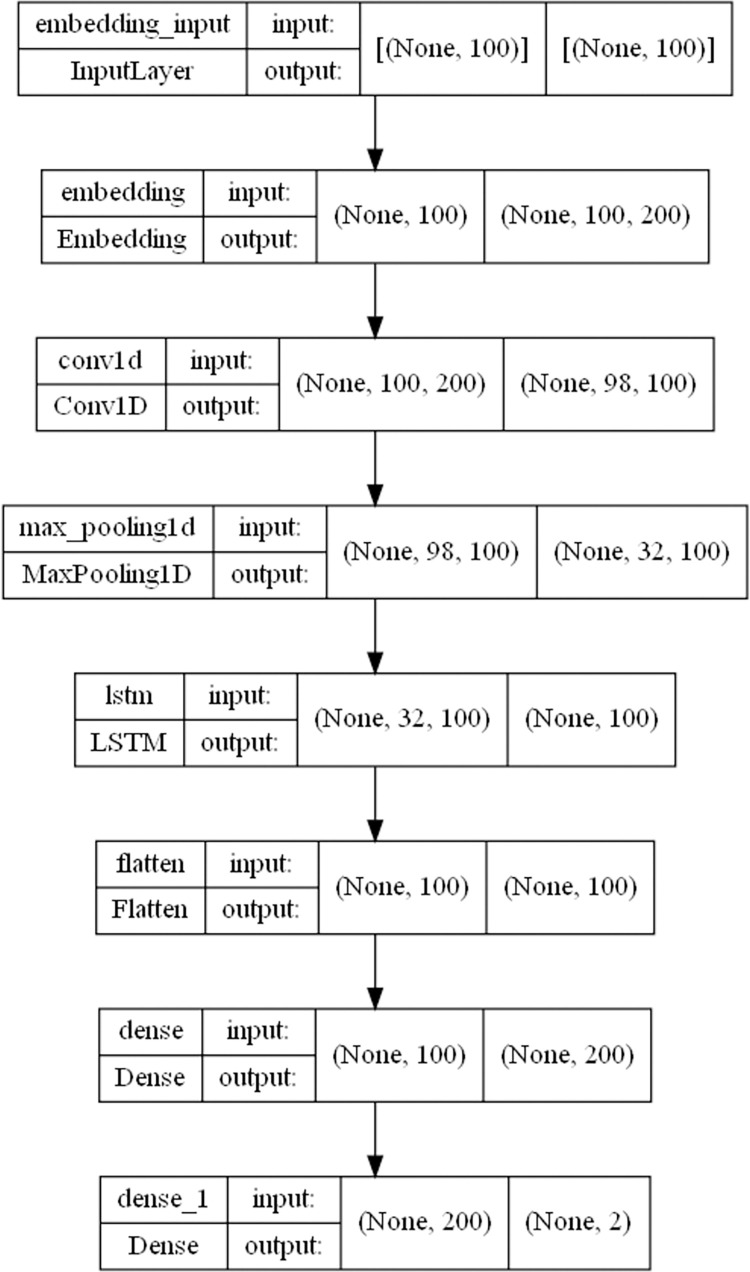
The analysis of sentiment by machine learning models.

In the third part of this study, we conducted sentiment analysis using machine learning models. Specifically, we utilized a CNN+LSTM model for sentiment recognition, which combined the feature extraction capabilities of CNN with the predictive analysis power of LSTM. Fig 7 illustrates the specific structure of the model, which begins with the input layer and progresses through the embedding layer, convolutional layer, maximum pooling layer, LSTM layer, fully connected layer, and output layer. We labeled the data with positive and negative sentiments in order to use supervised learning techniques. The labeled data was then split into a training dataset and a test dataset, and the trained machine learning model was used to perform predictive analysis on all the opinion data in the fourth part of the study.

This paper introduces an active learning strategy that was employed in the second round of experiments. The active learning intervention obviates the necessity for the annotation of the entirety of the data set, and the strategy can filter out some data for annotation, thereby effecting a substantial reduction in the costs incurred in terms of human and financial resources.

#### 4.1.2. Experiment and results

**Fig 8:**
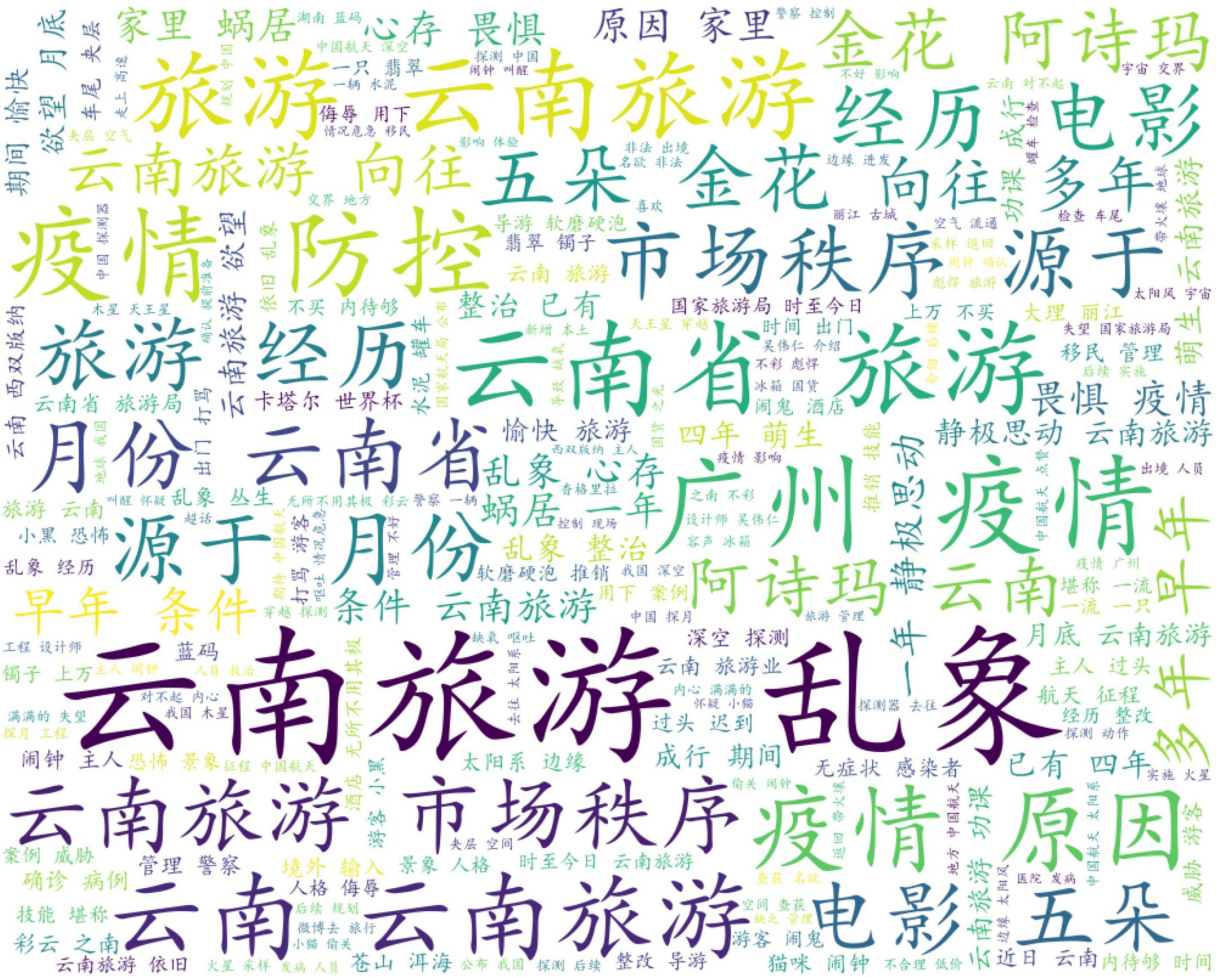
Word cloud graph.

**Fig 9:**
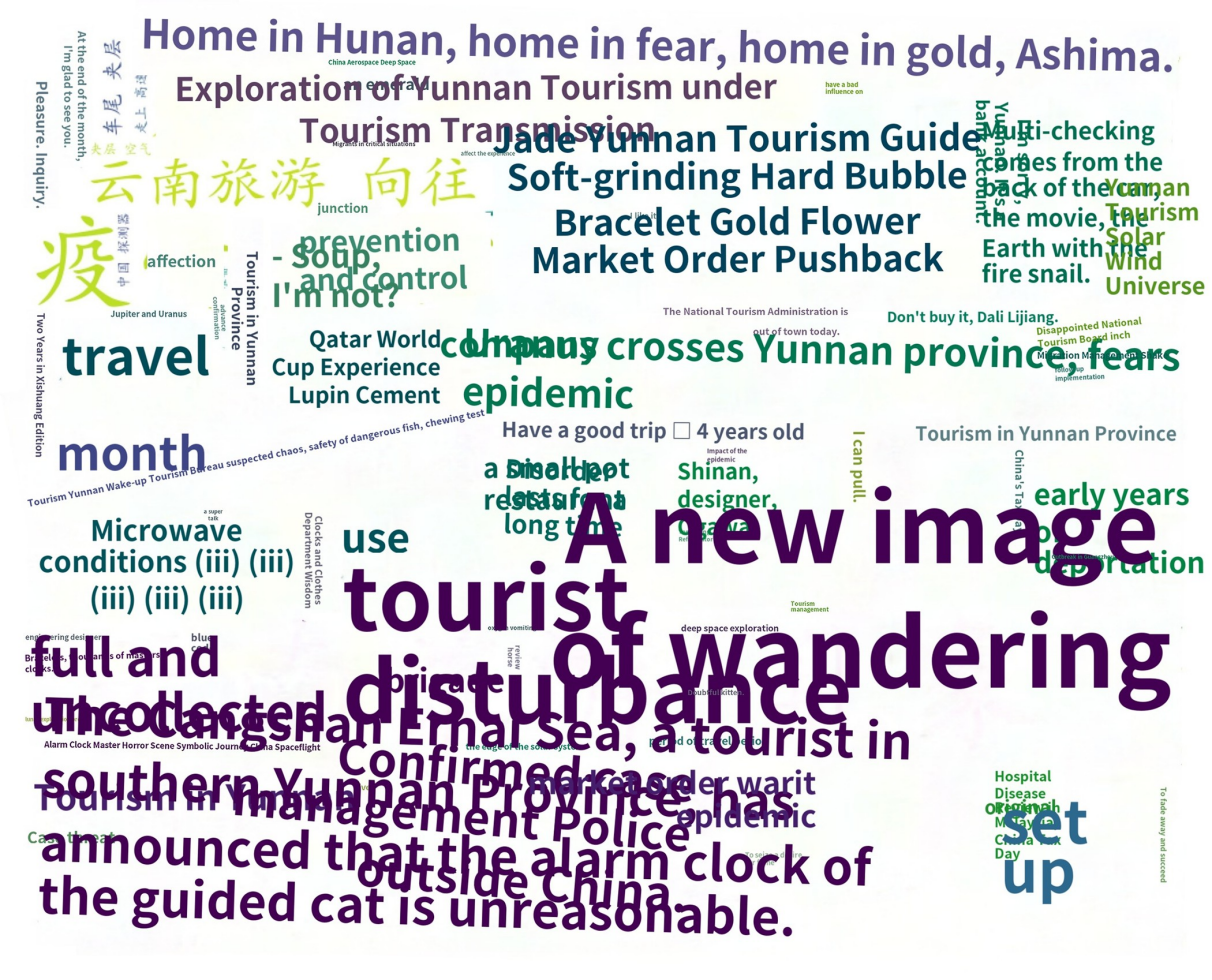
Translation of word cloud graph.

**Fig 10:**
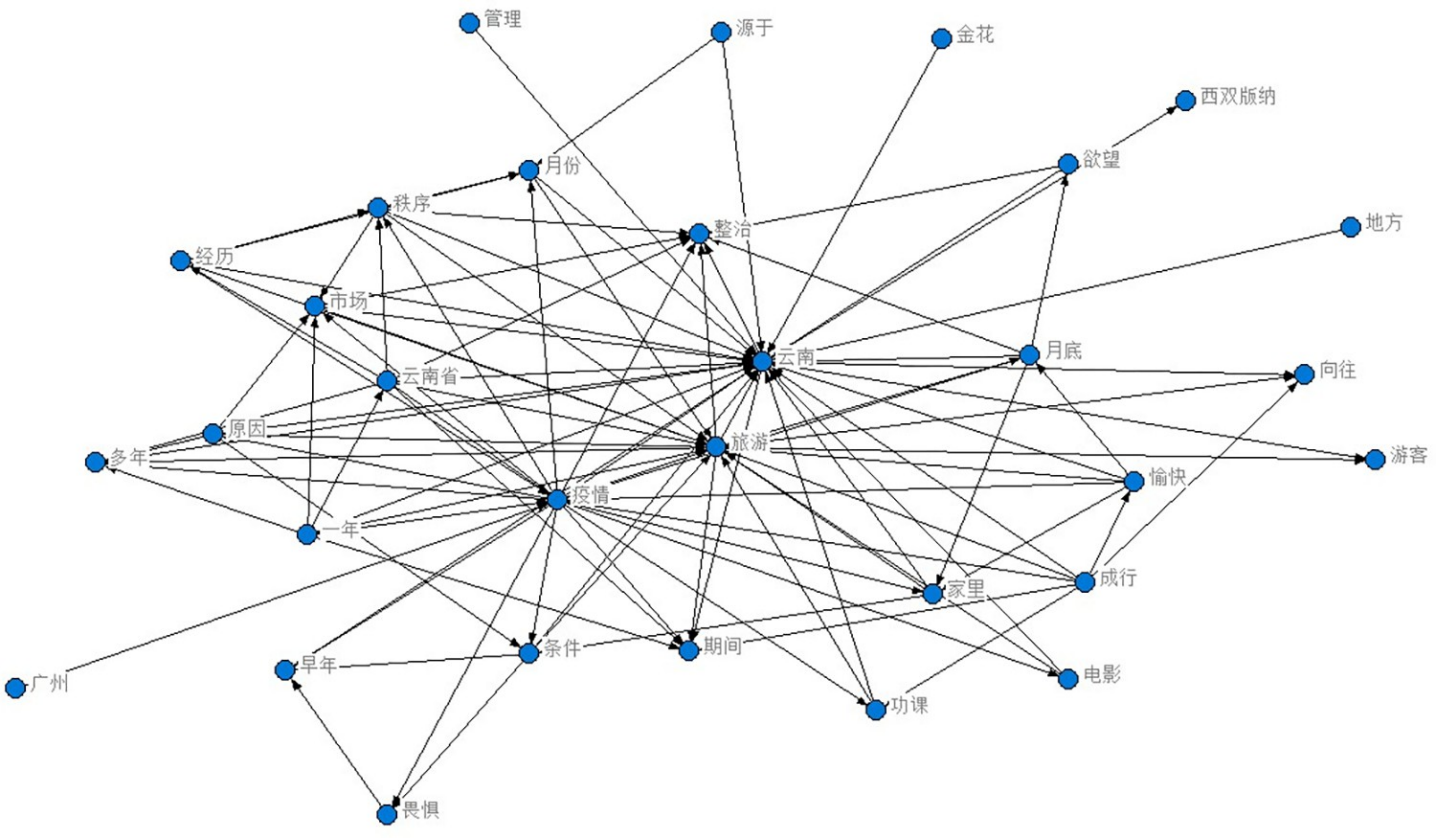
Semantic network graph.

**Fig 11:**
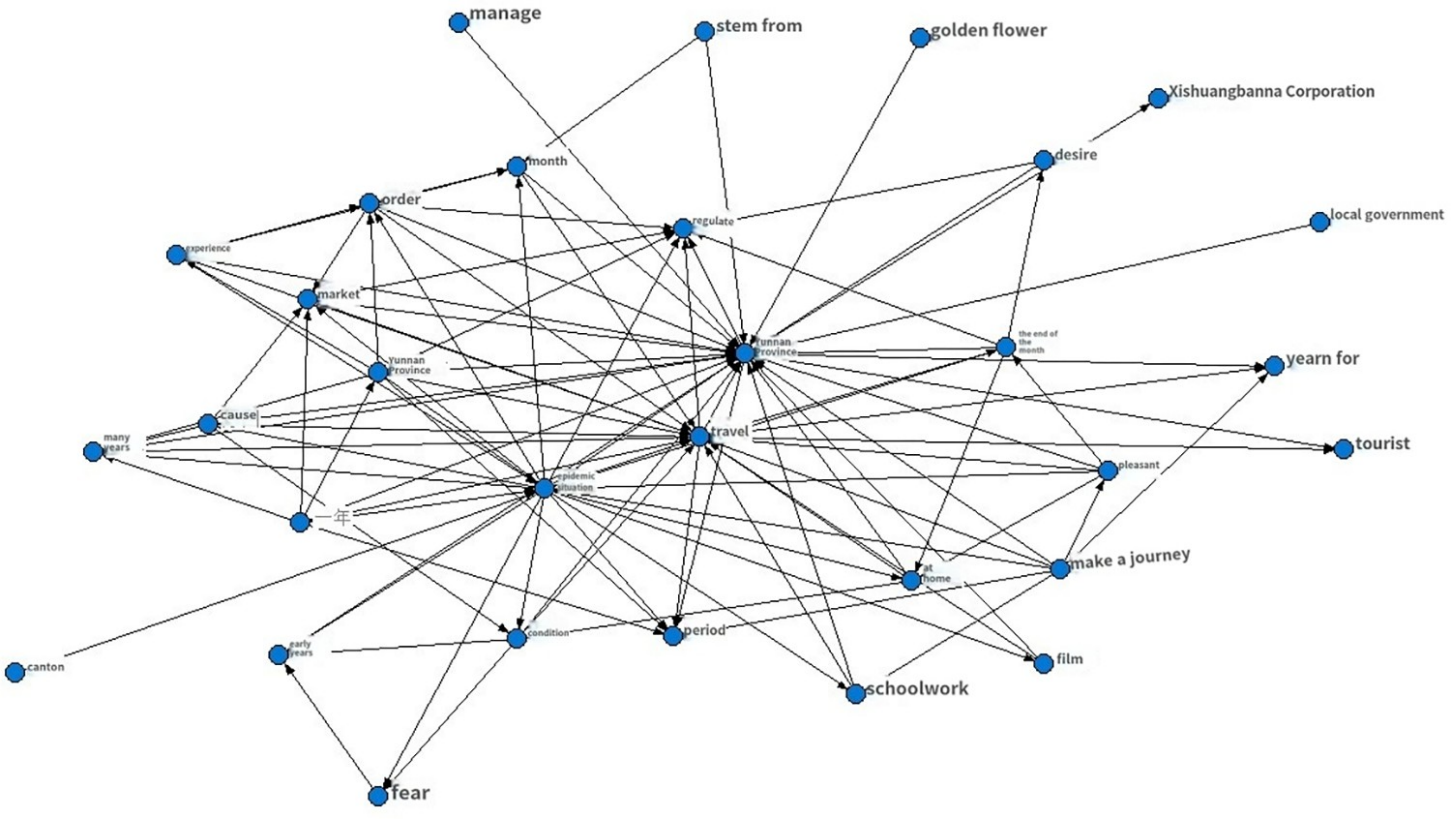
Translation of semantic network graph.

This study utilized crawler technology to gather blog posts and comments related to recent popular events, focusing on collecting data that included words specific to province Y, such as tourism. Approximately 40,000 pieces of data were collected, and after filtering by IP addresses located in provinces Y and G, 7899 pieces of data were obtained. Following the removal of missing and invalid data, 6519 pieces of valid data remained. Preprocessing techniques were applied to the data, resulting in the creation of a word cloud map. The ROST CM 6 (ROST Content Mining System Version 6.0) content mining system was then used to perform social network and semantic network analysis on the comment text, ultimately generating a semantic relationship map. As the data was in Chinese, a rough translation using PAPAGO’s [62] image recognition translation was employed to provide a general understanding of the results, as demonstrated in Figs 8–11. Fig 8 is the word cloud graph of text comments on Weibo. Fig 9 is the English-translated version of the image in Fig 8. Fig 10 is the semantic network graph of text comments on Weibo. Fig 11 is the English-translated version of the image in Fig 10. Figs 8–11 are Word cloud graphs and semantic network graphs, and their translation results. The direct translation of the images is not good, but still the main conclusions can be seen. This is the best translation software we found.

Based on the analysis of the data, it can be observed that tourism, chaos, and management are the most frequent topics of discussion, apart from those related to the COVID-19 epidemic. The words tourism, Yunnan (Province Y), tourists, experience, rectification, yearning, market, management, and beauty appear more frequently, and the statistical analysis has revealed that they are mutually linked. For example, the frequency of occurrence between tourism and Yunnan is 1606, between Yunnan and tourists is 703, between Yunnan and experience is 550, and between tourism and politics is 532. In the semantic network, “Yunnan”, "tourism", and "management" are the most closely related feature words, with the highest frequency of co-occurrence, making them the core words in the entire semantic network. These core feature words act as "bridges" in the semantic network, connecting various topics and discussions.

**Fig 12:**
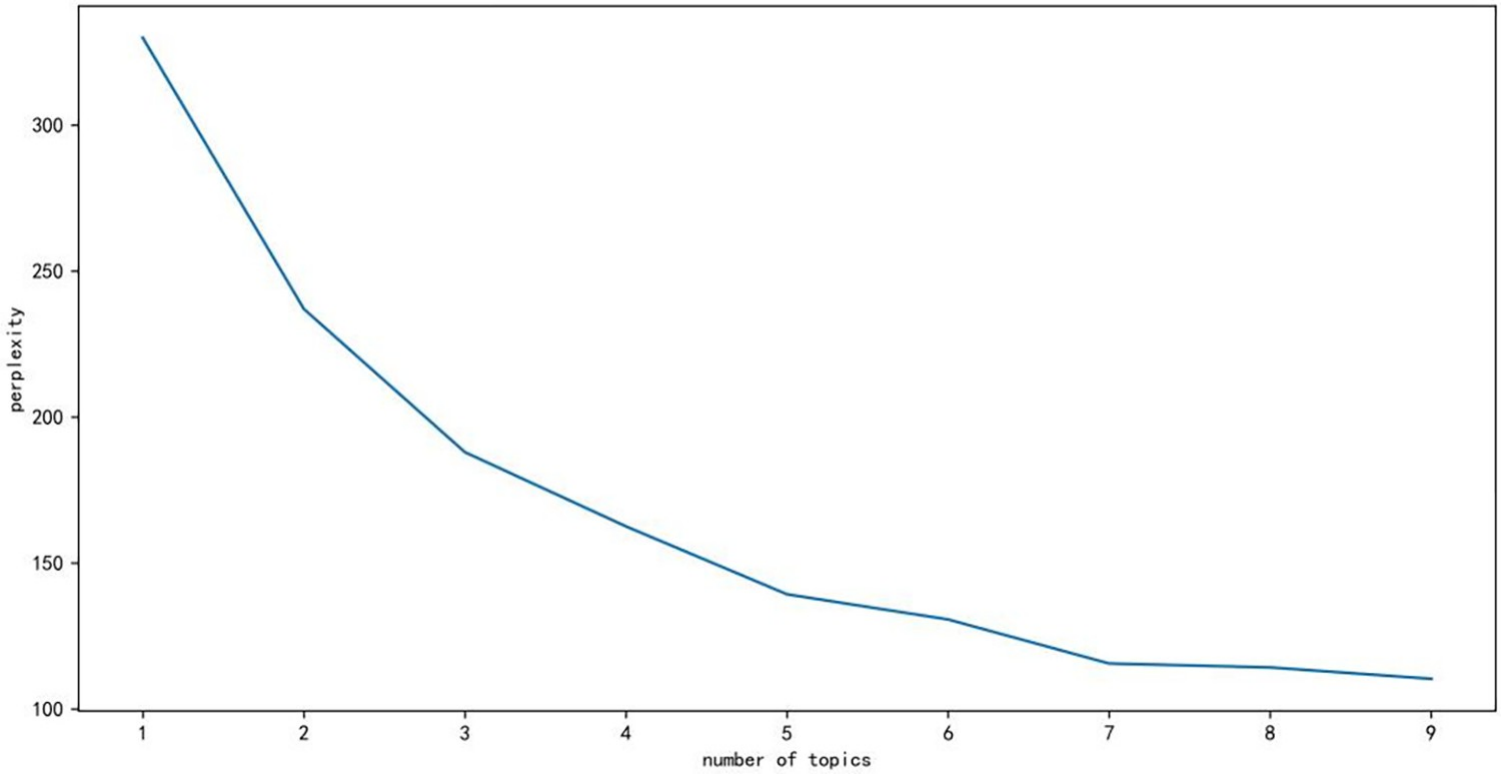
Perplexity-Topic line graph.

**Fig 13:**
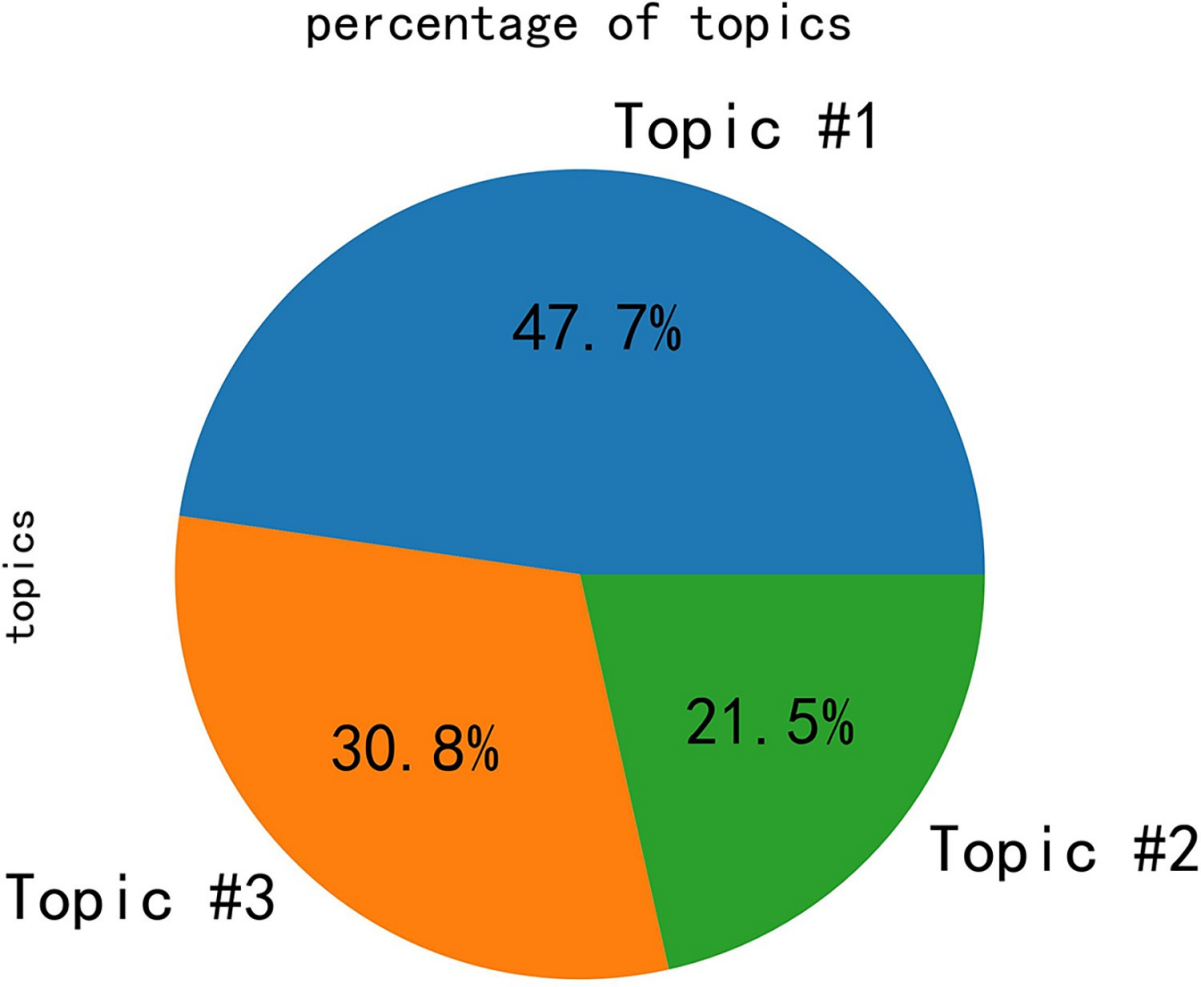
Distribution plots of topic strengths.

In the integrated semantic network, it can be observed that public opinion is primarily focused on the topics of tourism, experience, and management. Subsequently, the LDA algorithm was employed for the analysis of topic words, with the results depicted in Fig 12. Based on the graphical representation, the optimal number of topics for this study was determined to be 3. Subsequently, the LDA algorithm produced a distribution table of topic words, as shown in Table 1. Additionally, distribution plots of topic strengths were obtained, as illustrated in Fig 13.

**Table 1:** Distribution of topic words.

Topic 1	Probability	Topic 2	Probability	Topic 3	Probability
Province G	0.0395	ProvinceYTravel	0.0921	Province Y	0.0535
Outbreak	0.0392	Tour	0.0517	Tourists	0.0326
China	0.0210	Province Y	0.0375	Dali	0.0280
Cases	0.0157	Chaos	0.0334	Lijiang	0.0269
Preventionand control	0.0157	Province Y	0.0258	Province Y tour	0.0249
Code Blue	0.0156	Management	0.0152	Xishuangbanna	0.0224
Guangdong	0.0137	yearning	0.0144	Erhai	0.0223
ChinaAerospace	0.0128	Years	0.0137	Chaos	0.0217
Detection	0.0122	Remediation	0.0127	Shangri-La	0.0182
Baiyunshan	0.0121	outbreak	0.0127	Tour	0.0176
New cases	0.0117	at the end of the month	0.0126	alarm clock	0.0138
Wu Yifan	0.0113	home	0.0125	Kittens	0.0135
China	0.0106	reason	0.0125	Guided Tour	0.0120
confirmed	0.0092	a year	0.0121	Places	0.0107
Infectedperson	0.0085	movie	0.0121	Hotels	0.0104
Asymptomatic	0.0080	during	0.0120	Cats	0.0103
Beijing	0.0080	experience	0.0118	owner	0.0097
Hunan	0.0080	Market Order	0.0118	Colorful clouds	0.0093
Nucleic acid	0.0077	Conditions	0.0118	Time	0.0091
Local	0.0074	Already	0.0118	Really	0.0089

Certainly, the results of the study show that the embodiment of public opinion in the analyzed data roughly revolves around tourism, experience, and management in Province Y. The LDA algorithm identified three main topics: Topic 1, reflecting social hotspots such as the COVID-19 pandemic, nucleic acid testing, and spaceflight; and Topics 2 and 3, emphasizing the tourism situation in Province Y, with words related to tourism and management in the province. The core feature words of the integrated semantic network are "Yunnan," "tourism," and "management," which have the highest frequency of co-occurrence and serve as the "bridges" connecting the entire semantic network. Overall, the study highlights the importance of monitoring public opinion in the context of tourism management and suggests that social media analysis can provide valuable insights for tourism destination management.

This paper addresses various constraints associated with the proposed perceptual model, including data collection, real-time data, data fairness, and the interpretability of the model for public opinion issues. The data used in this study comes from China’s largest online social platform, Sina Weibo, and was collected in December 2022, consisting of approximately 40,000 real and real-time online comments. To facilitate the analysis, the perceptual model segments the collected data based on the IP address of the posted comments, resulting in approximately 8,000 data points. This segmentation is convenient for inferring public opinion and sentiment in both Province Y and Province G, which meets the requirements for training and testing related machine learning models. Importantly, the collected online comments are spontaneously formed by netizens around hot topics, and no different collection methods are applied to different groups. Therefore, the model results are fair and the model’s perception results are reliable. As for the management model used in this study, the evolutionary game model is widely recognized for studying complex system interactions and individual strategy evolution paths. Based on the analysis results of the perception model, this paper accurately identifies the pertinent issues requiring attention in cyberspace governance, as well as the development patterns of various stakeholders. By applying the evolutionary game model, the behaviors and evolutionary patterns of stakeholders in managing public opinion and sentiment can be accurately analyzed. As a result, the "perception management" model proposed in this paper is not only reliable in terms of data collection, real-time data, and fairness but also effective in the perception and management of public opinion.

After labeling the data with positive and negative sentiment, we split 70% of the data into a training set and 30% into a test set. The machine learning model was constructed using Keras, with a 100-dimensional word vector and a convolutional layer with a "relu" activation function. For the convolutional layer, the number of convolutional kernels was set to 100, and the window size of each kernel was set to 3 time steps. Using "relu" as the activation function is computationally efficient, provides low model complexity, and effectively addresses issues such as gradient vanishing and explosion during machine learning training.

To optimize the model, we used "categorical_crossentropy" as the loss function and "Adam" as the optimization algorithm. " categorical_crossentropy" is ideal for measuring the difference between the model output and the target label, which makes it suitable for sentiment classification in this study. "Adam (Adaptive Moment Estimation) is an adaptive gradient descent algorithm that adjusts the learning rate during training to help the model converge to the global optimal solution or a near optimal solution. This optimization technique speeds up training, saves time and computational resources, and reduces the likelihood of getting stuck in local optima.

Other hyperparameters were set based on conventional values used in related machine learning studies. In this paper, we set the learning rate to 0.0001, the number of training epochs to 20, and the batch size to 256. To combat overfitting problems, we introduced the dropout regularization technique after the convolutional layer, with a dropout ratio of 0.2. Dropout randomizes the output of some neurons, reducing the complexity and dependency in the neural network. This prevents certain neurons from overfitting the training data, improving the model’s generalization ability. In addition, the model implements an early stopping mechanism to address overfitting. By monitoring the model’s performance on the validation set during training, we stop the training process when the model’s performance stops improving or starts to decline. This ensures that the model does not overfit the training data and selects the model with optimal performance.

**Fig 14:**
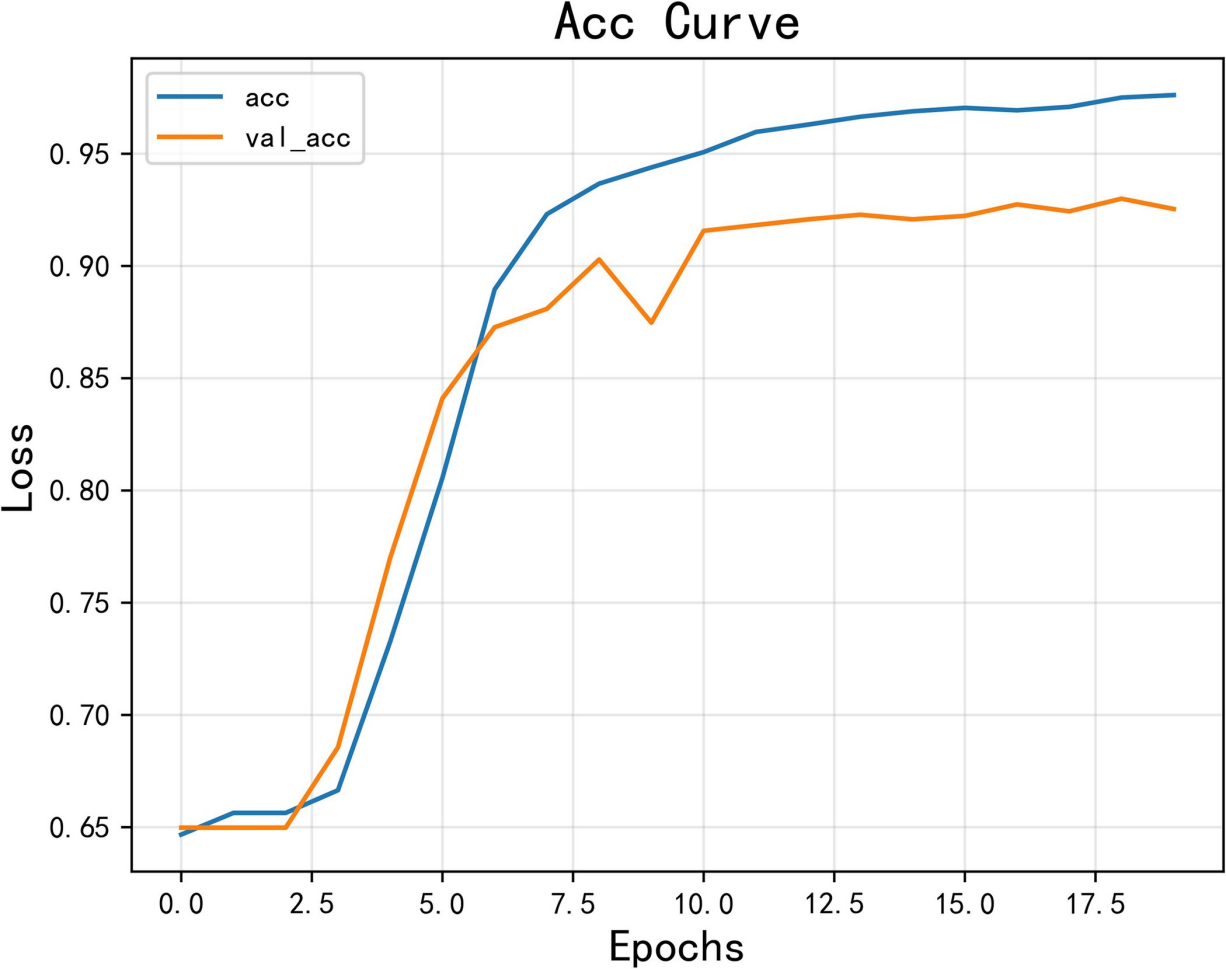
The variation curve of accuracy rate.

**Fig 15:**
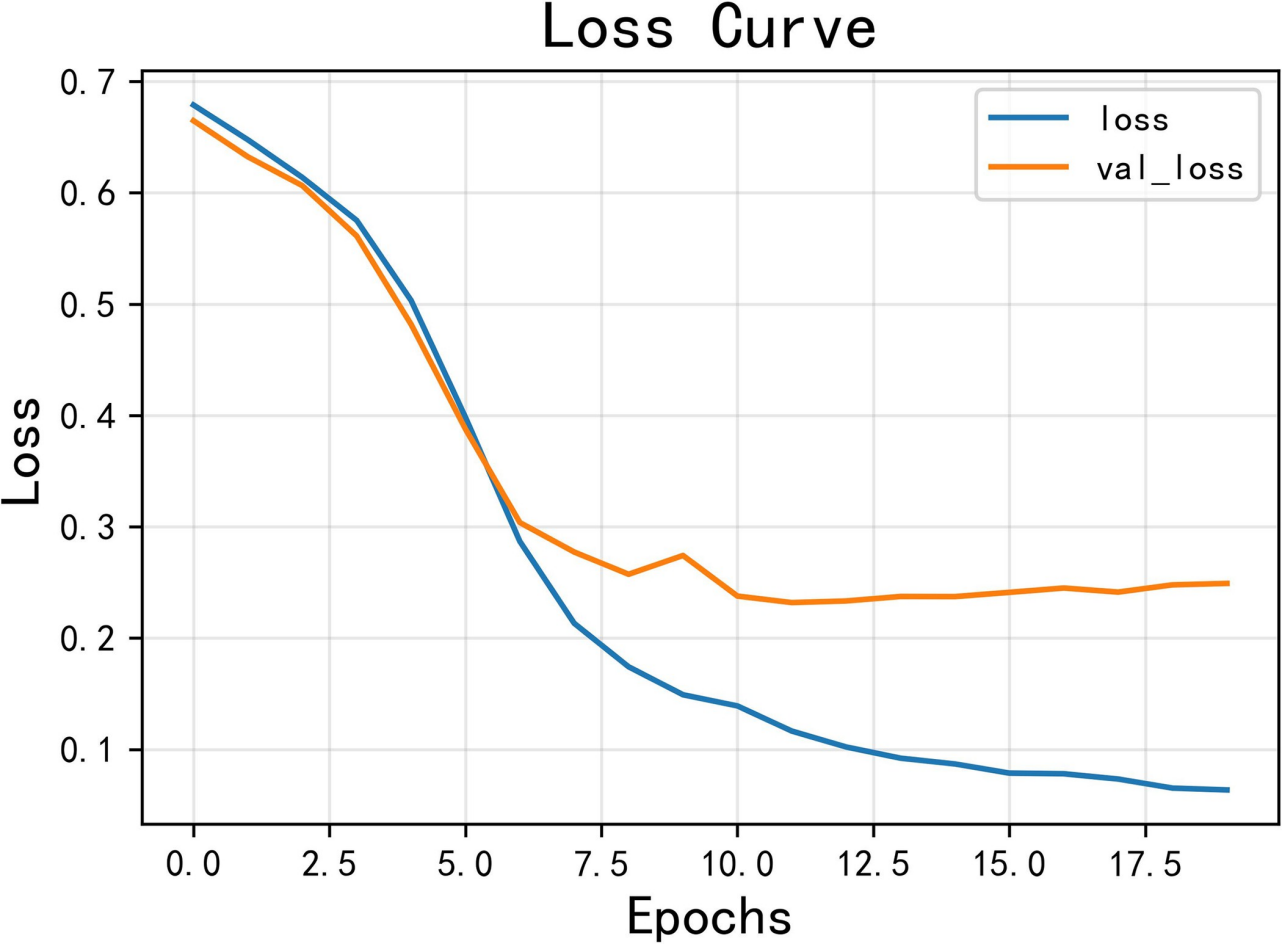
The variation curve of loss.

**Fig 16:**
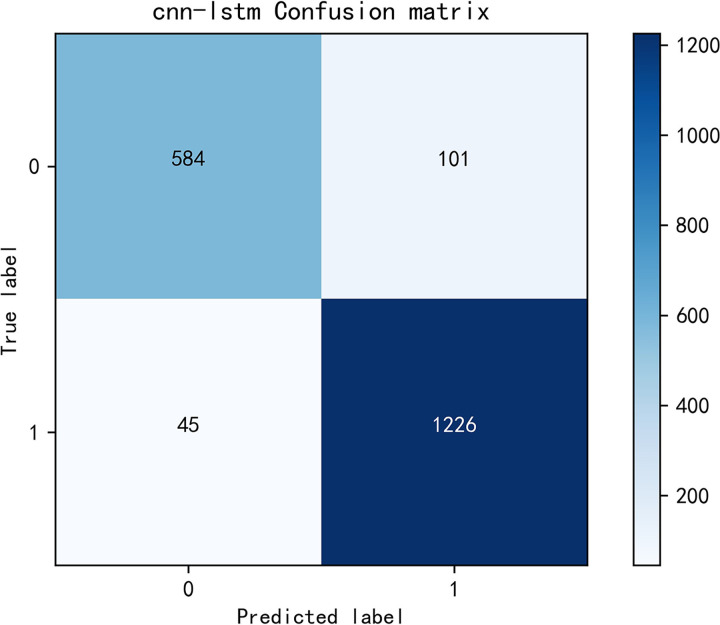
Confusion matrix.

The model achieved an accuracy of 0.925 and an F1 score of 0.924, as shown in Figs 14 and 15 for loss and accuracy over time and Fig 16 for the confusion matrix.

**Fig 17:**
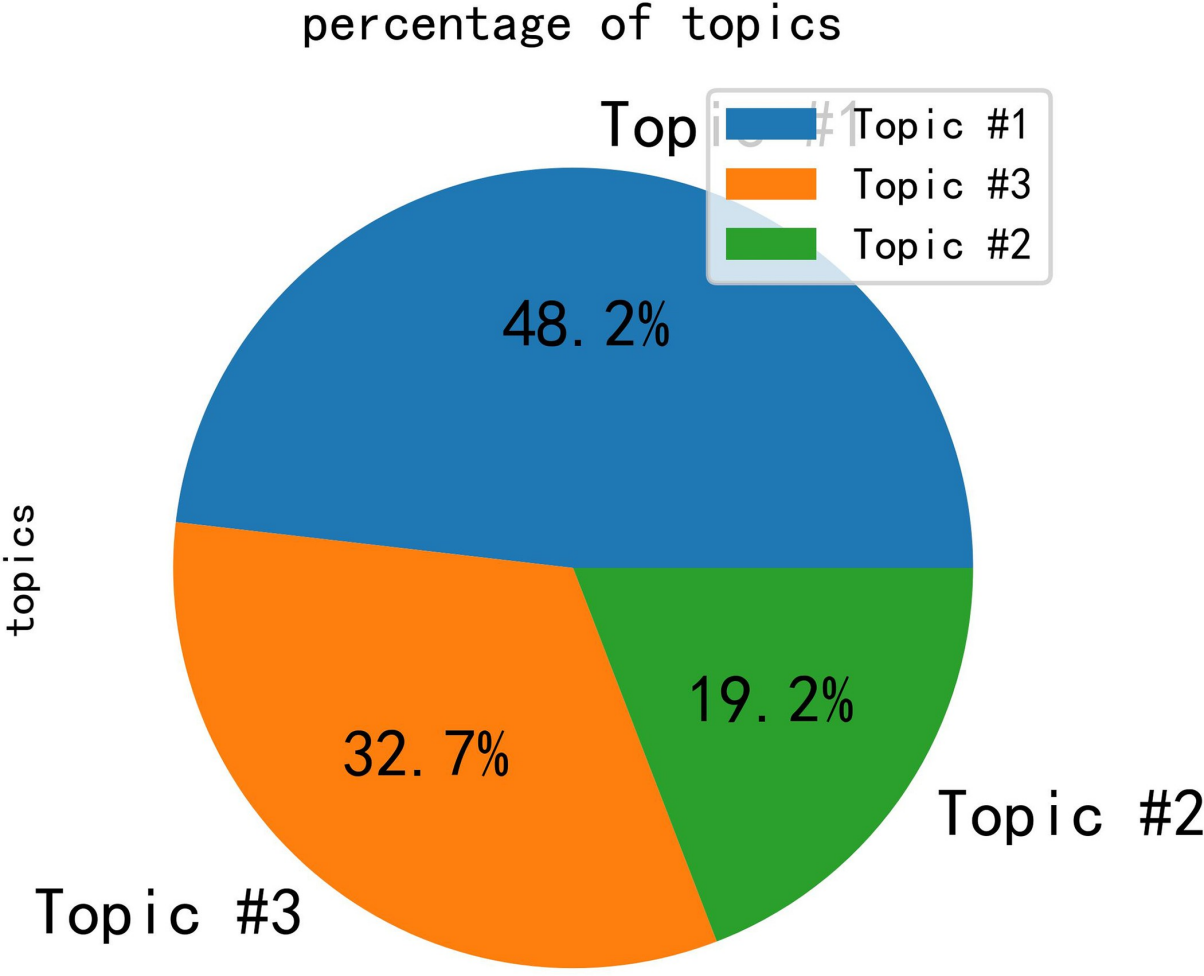
Positive sentiment graph.

**Fig 18:**
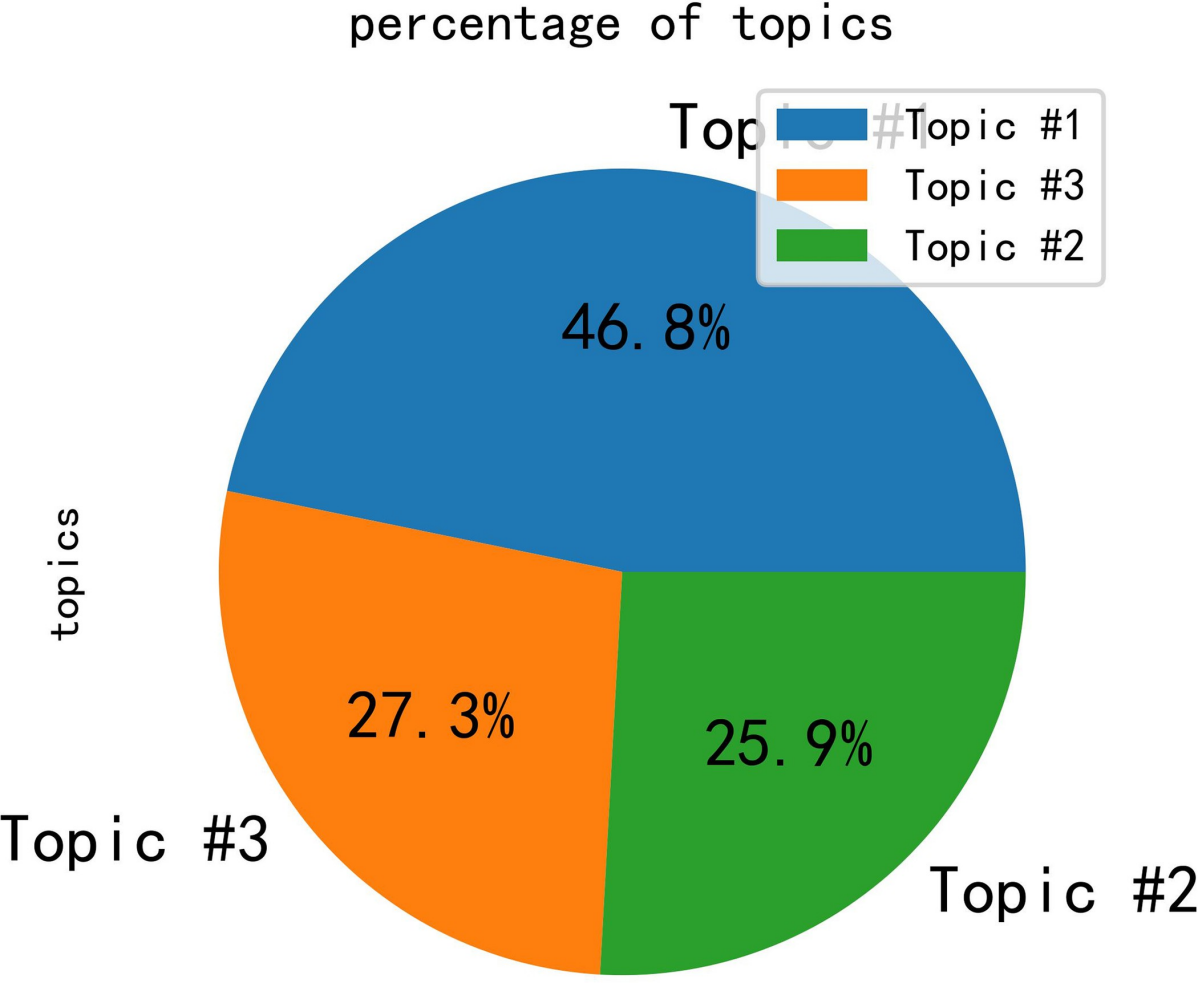
Negative sentiment graph.

The results of the analysis indicate that the negative sentiment share of Province Y is 0.333, while the negative sentiment share of Province G is 0.30. This suggests that the negative sentiment toward Province Y is 12% higher than that of Province G. The trained model was employed to obtain the positive sentiment and the negative sentiment share, as illustrated in Figs 17 and 18. This information may prove useful in the formulation of decisions and policies related to the management of both provinces. Figs 17 and 18 depict the topic intensity distribution chart, which illustrates the degree of influence a given topic exerts on the overall sentiment.

**Fig 19:**
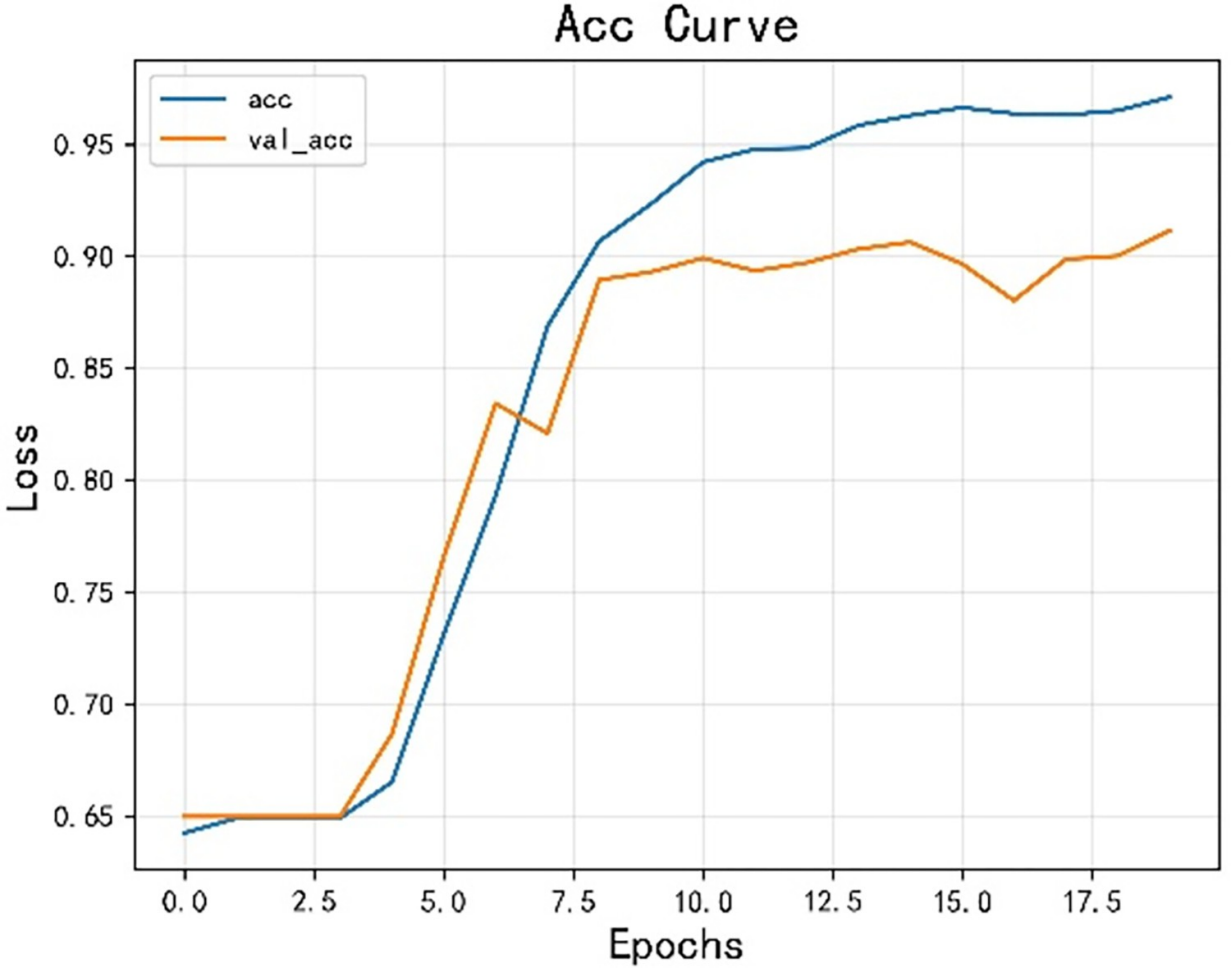
Change curve of accuracy.

**Fig 20:**
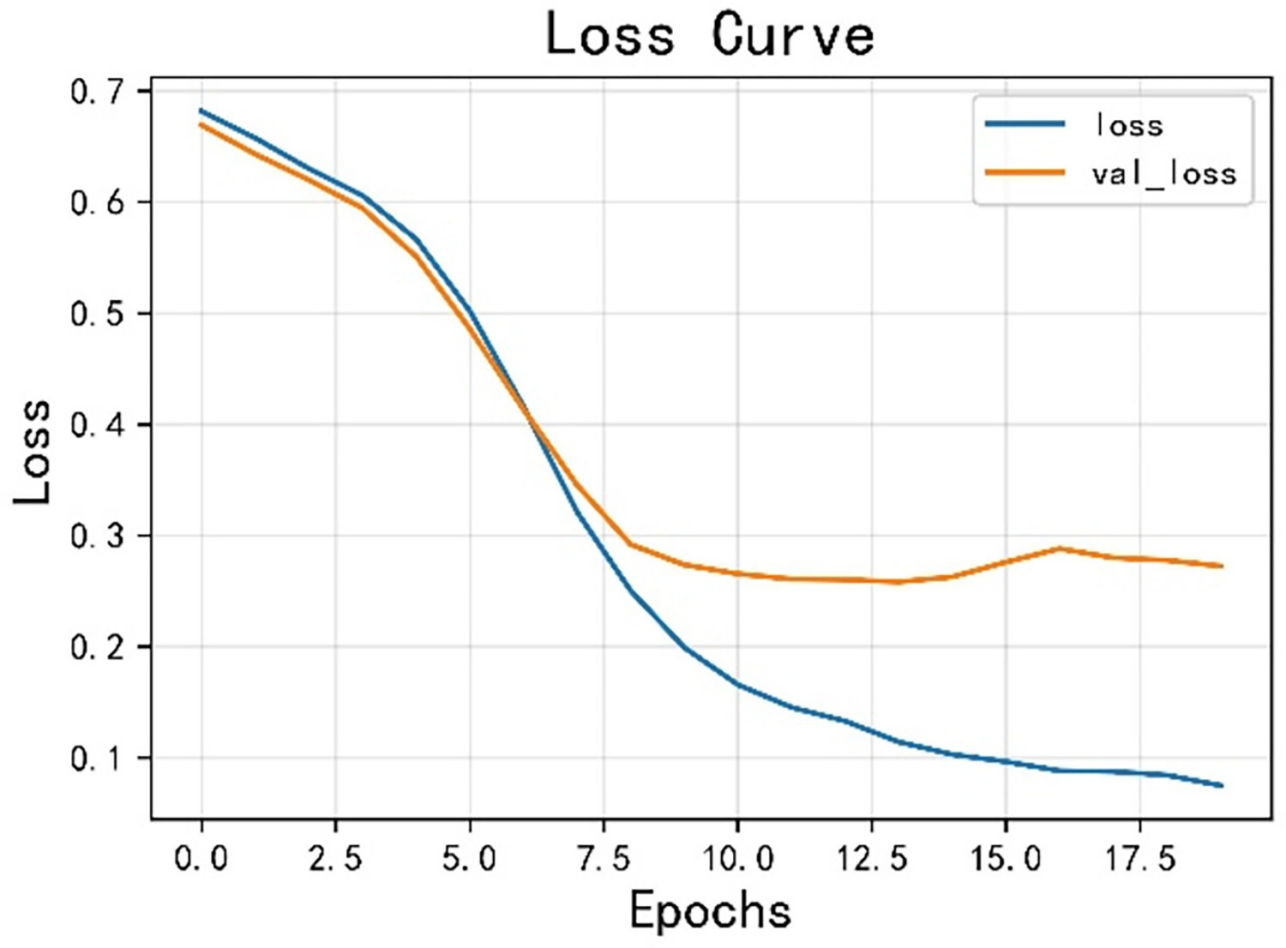
Change curve of loss.

**Fig 21:**
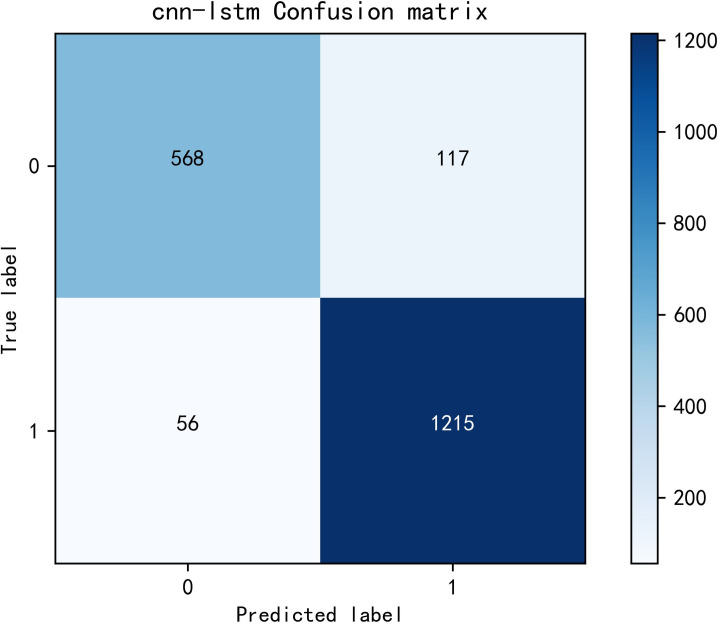
Confusion matrix.

In the second round of experiments, we implemented an active learning strategy with the only difference being the reduction of the training data. Since we are only focusing on verifying the effectiveness of active learning and not discussing the optimization of the strategy, we used the traditional active learning strategy ’random’ in this study. Specifically, we left 20% of the data unlabeled. The results, shown in Figs 19–21, reveal an accuracy of 0.911, which is only 1.53% lower than the previous model, and an *F*_1_ score of 0.910, which is only 1.54% lower. These findings demonstrate that the active learning strategy is fully applicable and can significantly reduce costs in this type of situation analysis. Figs 19–21 are model training results after the introduction of active learning.

The opinion perception model proposed in this paper excels in sentiment analysis. With the incorporation of active learning, the performance of the model experiences a slight decrease, but it remains well within the accuracy requirements for opinion analysis specified in this paper. Importantly, this incorporation significantly reduces the cost, making it a valuable enhancement. Related studies are using CNN and LSTM for sentiment analysis, this paper uses a combination of CNN and LSTM, so the model proposed in this paper is compared with CNN and LSTM [63, 64]. Table 2 shows a comparison of the proposed model with CNN and LSTM algorithms.

**Table 2:** Comparison table for models.

	CNN	LSTM	Proposed model	Proposed model with active learning
Accuracy	0.853	0.887	0.925	0.911
F1 score	0.850	0.883	0.924	0.910

#### 4.1.3. Analysis

We present a framework for the perception and analysis of online public opinion, which allows for an effective examination of this topic. Our analysis revealed that, despite a decrease in social hotspots for tourism during the pandemic, the issue of tourism chaos was more severe in Province Y. Furthermore, negative sentiment towards tourism and regulation was more prominent in Province Y than in Province G. This suggests that online opinion has the potential to influence offline behavior. Furthermore, the active learning strategy was found to be highly applicable in this context. Despite employing a straightforward active learning approach (random), we attained favorable outcomes, with an accuracy of 0.911 and an F1 score of 0.910. By reducing the training data by 20%, our framework demonstrates the ability to conserve resources without sacrificing performance. The findings of our study corroborate the efficacy of our framework and substantiate the capacity of active learning to facilitate the analysis of online public opinion.

### 4.2. Relationship between popular will, internet public opinion, and people’s behavior

This section discusses the hierarchical relationship between popular will, internet public opinion, and people’s behavior, as well as the interactions between them.

While the term "popular will" has been used for a long time, it remains an ambiguous term in the social sciences, drawing from multiple disciplines such as political science, sociology, and psychology. Traditionally, popular will has been interpreted as public opinion, reflecting the idea that the power of the state is derived from the people and that popular will possesses the power of law and is irrevocable. In essence, popular will encompasses a range of meanings, including the opinions of individuals, groups, and non-governmental institutions, and may be expressed publicly or privately. However, expressing public opinion does not necessarily indicate a tendency to act or lead to a public event.

An "Internet trending event" is defined as an event that receives extensive coverage on the Internet, resulting in a high level of engagement and discourse among online users. These events are frequently associated with contemporary social concerns or the activities of prominent individuals, and are disseminated via a range of online platforms, including news websites, social media platforms, and microblogs. Internet trending events typically elicit a high level of engagement, as evidenced by high click-through rates, likes, shares, reposts, and comments. This reflects the public’s interest and emotional response to these topics. In this manner, trending topics function as a significant indicator of contemporary social trends and public sentiment.

**Fig 22:**
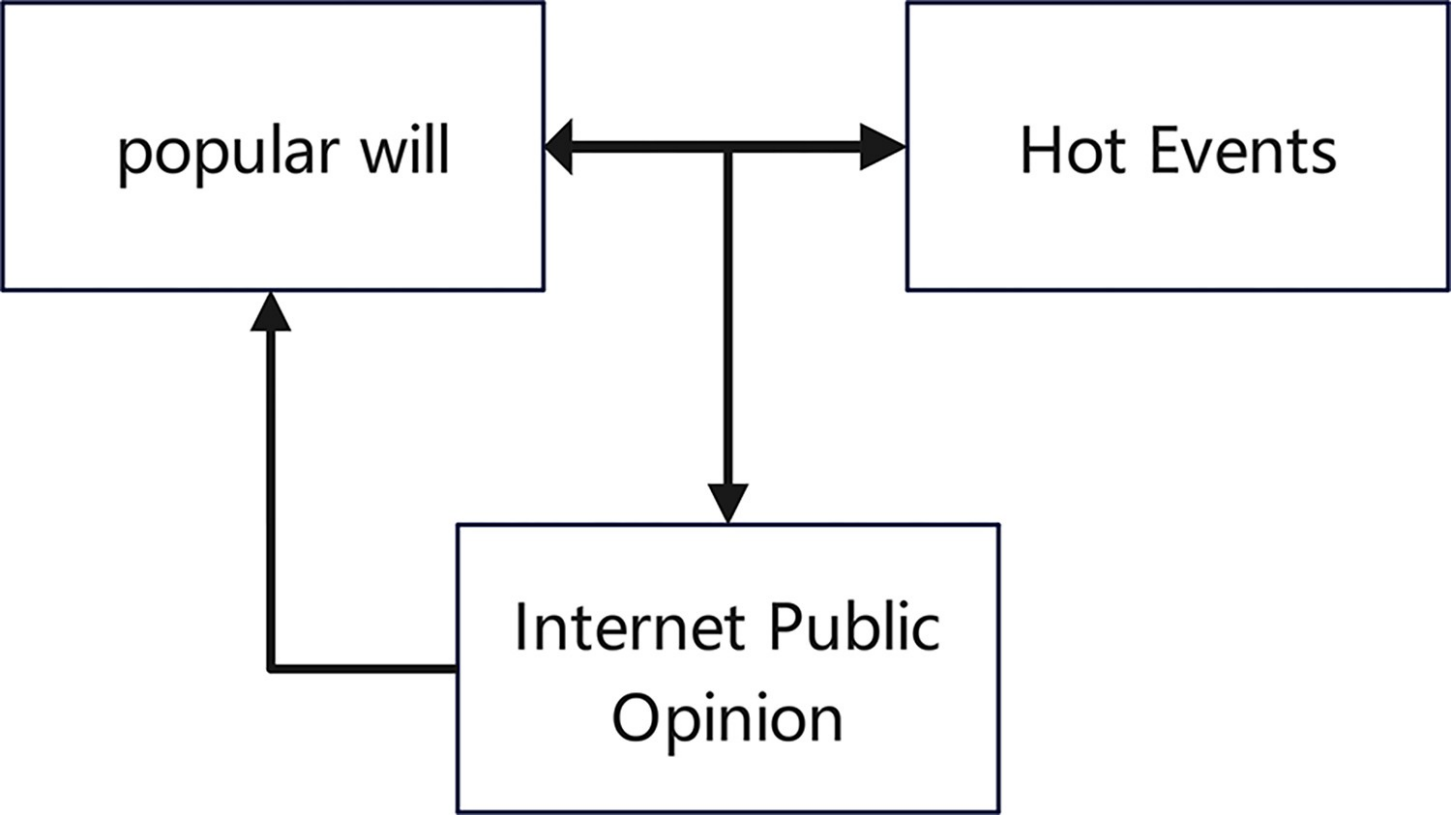
The relationship of popular will, hot events, and internet public opinion.

The relationship between popular will, internet public opinion, and hot events can be illustrated in Fig 22. Popular will is a pre-existing and potential public sentiment that may not be directly related to the occurrence of hot events. However, when relevant events happen, the internet public will use the internet platform and channels to express, spread, and communicate their opinions on the events. This will, in turn, influence the progress and direction of the events, and the development of the events will also impact the original popular will. The occurrence of internet public opinion is eventually catalyzed under their influence. Hot events are widely disseminated on the internet, attract the common attention of internet users, stimulate the participation of the internet community, and form public opinion to drive the process. The power of public opinion in the network may influence the development and direction of the event, which is the formation of public opinion in a hot event.

Based on the above analysis, apparent public opinion can be defined as the expression and manifestation of potential public opinion through the Internet platform, which is characterized by publicity and visibility. Through the rapid dissemination and amplification of the Internet, potential public opinion is transformed into apparent public opinion, which further influences people’s behavior. Apparent public opinion not only reflects people’s emotions and attitudes toward certain events or issues, but also guides and influences people’s behavior. In the era of Internet public opinion, people’s behavior is no longer limited to offline activities, but also includes online behaviors such as commenting, sharing, and liking, which are all manifestations of apparent public opinion. Therefore, the relationship between public opinion and behavior in the Internet public opinion era is complex and intertwined, with both potential and apparent public opinion shaping and influencing people’s behavior.

**Fig 23:**
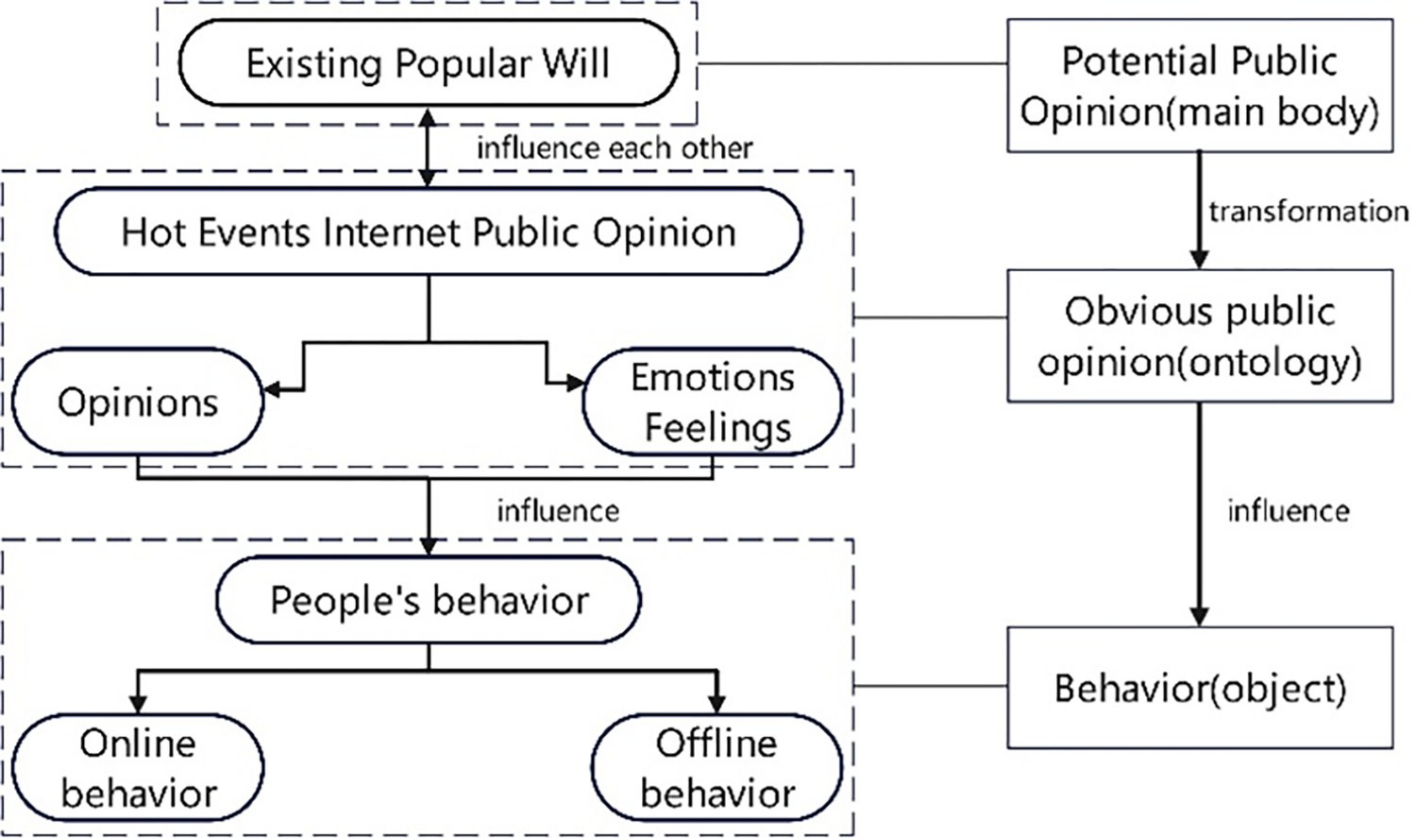
The relationship between popular will, internet public opinion, and behavior.

Based on the Fig 23, we can see that popular will serves as a foundation and potential source of public opinion, which can be triggered by hot events and further develop into internet public opinion through online communication and interaction among netizens. Internet public opinion, in turn, can influence people’s attitudes and behaviors both online and offline, creating a feedback loop with popular will. This relationship highlights the importance of understanding and managing public opinion, especially in the era of the internet, where information spreads rapidly and widely, and public opinion can have a significant impact on society and individuals.

This statement underscores the pivotal role of internet public opinion in the resolution of social conflicts. By ensuring that online public opinion is oriented towards positive outcomes, such as responding to the concerns of netizens, resolving doubts and confusion, and promoting scientific decision-making by the government, internet public opinion can help to release social pressure and stabilize social order. Moreover, by optimizing cyberspace and advancing sustainable human development, internet public opinion can contribute to the formation of a more positive and harmonious society. It is therefore imperative that measures be taken to guarantee that online public opinion is well-informed, constructive, and guided by a robust sense of social responsibility.

Based on the results of the first part of the study, it is evident that the issues of tourism chaos and management problems in Province Y have always been present as potential public opinion or popular will, regardless of the impact of Covid-19. When a specific issue related to tourism is discussed, these pre-existing opinions begin to ferment and result in the formation of Internet public opinion, including opinions and emotions. The already formed Internet public opinion, in turn, reacts back to the popular will, forming a mutually influential relationship. In other words, the negative perception of the tourism market in province Y has always existed, and under a certain topic, it amplifies and creates unfavorable Internet opinions that hinder the development of tourism in the region. This, in turn, influences people’s perceptions and behaviors both online and offline, leading to a negative evaluation of tourism in Province Y and consequently discouraging others from visiting the region.

Consequently, the relationship between popular opinion and internet public opinion has been subjected to analysis, resulting in the conclusion that the management of tourism in Province Y is in need of improvement. The initial section of the study, which focuses on the theme of "management," underscores the necessity for enhanced managerial techniques within the tourism sector in Province Y.

### 4.3. Game theory modeling analysis of public opinion management in Y province

In our previous section, we successfully used the perception model to accurately analyze the public opinion landscape in Province Y and Province G. Through this analysis, we identified the need for public opinion management in Province Y. Building on the "perception management" framework proposed in this paper, we used game theory tools to model and analyze the management of public opinion in Province Y. Evolutionary games are a refinement and extension of traditional games, addressing the limitations of traditional game theory [65]. Evolutionary games combine game theory and dynamic evolutionary processes, emphasizing dynamic processes rather than focusing on the static, and explaining the reasons and ways in which players reach a steady state. By applying the game theory tools, we gained a deeper understanding of the interrelationships and interactions among the various stakeholders. By applying the analytical tools of game theory, we gained a deeper understanding of the interrelationships and interactions among the various stakeholders, thus enabling the development of a more robust and scientifically based public opinion management program. This process involved analyzing the stakeholders and their behavioral choices, which then informed the public opinion management strategies. This management aspect of the perception management framework uses the insights gained from the perception model analysis to provide practical applications and strategies for managing public opinion and thereby influencing people’s offline behavior. The introduction of the management model in this paper significantly enhances the overall practicality of the framework.

#### 4.3.1. Model building

The game model can be used to analyze the interaction and strategic behaviors of the three parties, and to find the optimal solution that benefits all parties. Specifically, the government needs to take effective measures to manage tourism in province Y, while the media needs to report objectively and accurately on the situation and provide guidance to the public. Internet users, on the other hand, should express their opinions and concerns in a constructive manner, rather than spreading negative rumors and misleading information.

Through the game model, the government can adopt a proactive strategy to promote the positive aspects of tourism in province Y, while addressing the negative factors and improving the management of tourism. The media can act as a mediator between the government and the public, and provide balanced and objective coverage of tourism issues. Internet users can participate in the discussion and provide feedback to the government and media, thus promoting the healthy development of internet public opinion.

In conclusion, the three-party game model proposed in this study provides a new perspective for managing internet public opinion, and can help to promote social stability and sustainable development. By balancing the interests of the government, media, and people, the model can effectively address conflicts and challenges in the field of public opinion on the internet.

The government can choose to manage or not to manage, and the probability of the government managing is P_1_. If the government chooses to manage, then the cost is C_1_, which corresponds to getting the management gain R_1_. At this time the people all get the gain of cyberspace governance V_31_. If the media chooses to promote, then they receive the loss from government management L_21_. If the government does not manage, then there is no cost, but they get the loss from negative internet opinion loss L_1_, and at this point if the people participate, they receive the loss of disappointment L_3_.

The media can choose to push or not push, and the probability of choosing to push is P_2_. if push, then you can get the traffic, click rate, and other gains R_2_, but have to pay the work cost C_2_. At this time if the people participate, will also gain attention and other gains V_32_. if the online media choose not to push, the people participate in the case, it will make the people feel bored, there will be fewer customers and other losses W_23_.

People can choose to participate or not to participate, the probability of participation is P_3_. If they participate, they can get the sense of participation and other income R_3_, to pay time, network fees, and other costs C_3_, if they do not participate there is no cost.

**Fig 24:**
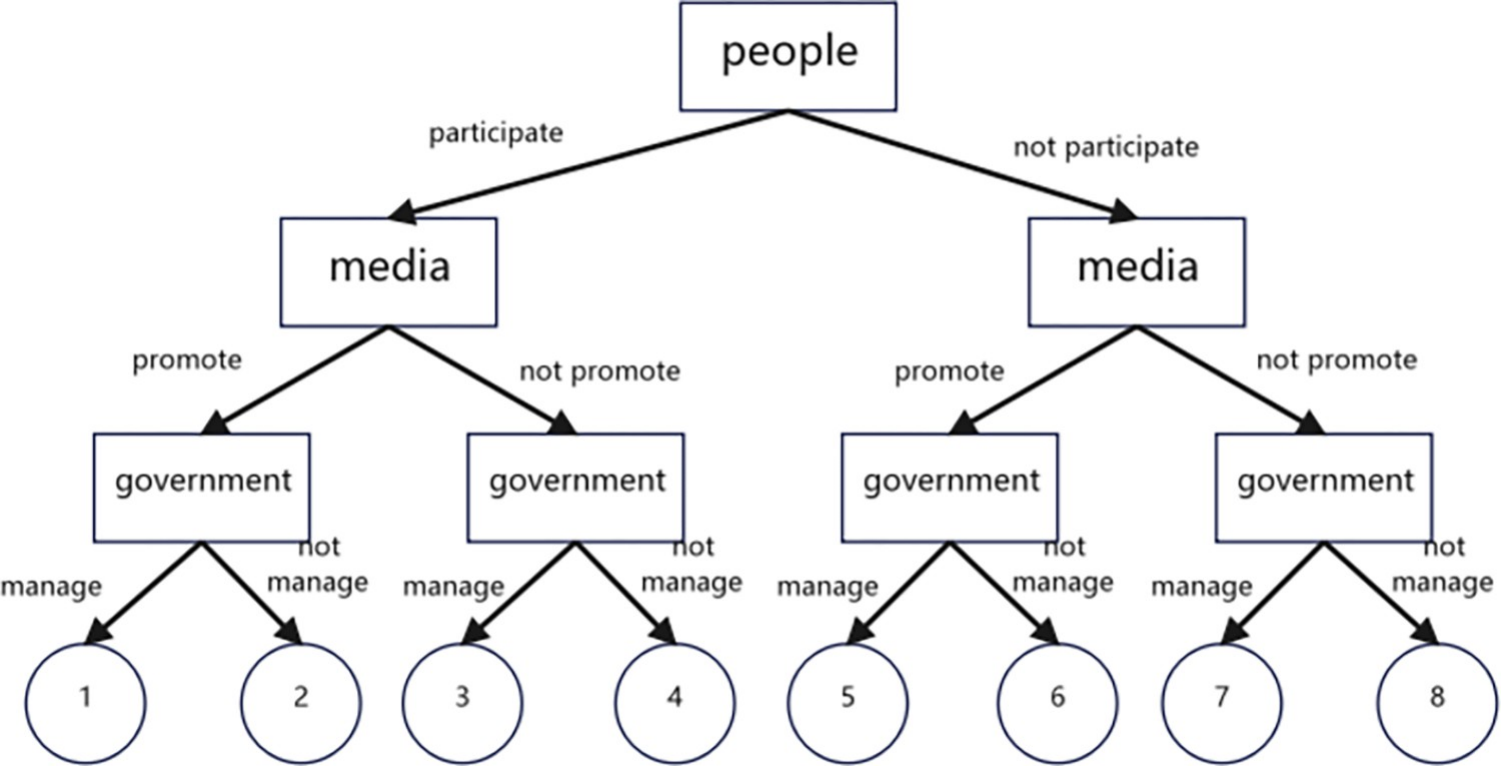
Government-media-people tripartite game tree.

Through the above concept definition and model assumptions, the government-media-people game model is established. In this paper, a game tree is used to represent the game process, as shown in Fig 24.

Based on the model assumptions and the game tree, the income matrix of government, media and people matrix, is shown in Table 3. The game strategies and benefits in this table results correspond to government, media, and people, respectively.

**Table 3:** Income matrix of government, media and people.

The strategies	The income
(manage, promote, participate)	$\left(R_{1}-C_{1}+L_{21},R_{2}-C_{2}-L_{21},R_{3}-C_{3}+V_{32}+V_{31}\right)$
(manage, promote, not participate)	$\left(R_{1}-C_{1}+L_{21},-C_{2}-W_{21},V_{31}\right)$
(manage, not promote, participate)	$\left(R_{1}-C_{1},-W_{23},R_{3}-C_{3}+V_{31}\right)$
(manage, not promote, not participate)	$\left(R_{1}-C_{1},0,V_{31}\right)$
(not manage, promote, participate)	$\left(-L_{1},R_{2}-C_{2},R_{3}-C_{3}+V_{32}-L_{3}\right)$
(not manage, promote, not participate)	$\left(-L_{1},-C_{2},0\right)$
(not manage, not promote, participate)	$\left(-L_{1},-W_{23},R_{3}-C_{3}-L_{3}\right)$
(not manage, not promote, not participate)	(−*L*_1_,0,0)

#### 4.3.2. Analysis of the model

1. Based on government management

Suppose that the manage of government is G_11_, the not manage of government is G_12_, so:

$$G_{11}=P_{2}P_{3}(R_{1}-C_{1}+L_{21})+P_{2}(1-P_{3})(R_{1}-C_{1}+L_{21})+(1-P_{2})P_{3}(R_{1}-C_{1})+(1-P_{2})(1-P_{3})(R_{1}-C_{1})=R_{1}-C_{1}+P_{2}L_{21}$$ (6)

$$G_{12}=-L_{1}P_{2}P_{3}+(-L_{1})P_{2}(1-P_{3})-L_{1}(1-P_{2})P_{3}-L_{1}(1-P_{2})(1-P_{3})=-L_{1}$$ (7)

Then the replication dynamic equation for the government’s choice of management as a strategy is,

$$F(P_{1})=\frac{dP_{1}}{dt}=P_{1}(1-P_{1})(R_{1}-C_{1}+P_{2}L_{21}+L_{1})$$ (8)

When $P_{2}=\frac{-R_{1}+C_{1}-L_{1}}{L_{21}},\;F(P_{1}\equiv 0)$, which means that all cases are evolutionary steady states. When $P_{2}\neq \;\frac{-R_{1}+C_{1}-L_{1}}{L_{21}}\;$, let F(P_1_) = 0, which gives P_1_ = 0 and P_1_ = 1 as the two equilibrium points. Finding the derivative of F(P_1_), it follows that,

$$\frac{dF(P_{1})}{dP_{1}}=(1-2P_{1})(R_{1}-C_{1}+P_{2}L_{21}+L_{1})$$ (9)

When $P_{2}>\frac{-R_{1}+C_{1}-L_{1}}{L_{21}},\;P_{1}=1$ is the equilibrium point and government manage is the evolutionary stable strategy; When $P_{2}<\frac{-R_{1}+C_{1}-L_{1}}{L_{21}},\;P_{1}=0$ is the equilibrium point and government not manage is the evolutionary stable strategy.

Based on media promote

Suppose that the promote of media is M_11_, the not promote of media is M_12_, so:

$$M_{11}=P_{1}P_{3}(R_{2}-C_{2}-L_{21})+P_{1}(1-P_{3})(-C_{2}-L_{21})+(1-P_{1})P_{3}(R_{2}-C_{2})+(1-P_{1})(1-P_{3})(-C_{2})=P_{3}R_{2}-P_{1}L_{21}-C_{2}$$ (10)

$$M_{12}=P_{1}P_{3}(-W_{23})+(1-P_{1})P_{3}(-W_{23})=-P_{3}W_{23}$$ (11)

Then the replication dynamic equation for the media’s choice of promote as a strategy is,

$$F(P_{2})=\frac{dP_{2}}{dt}=P_{2}(1-P_{2})(P_{3}R_{2}-P_{1}L_{21}-C_{2}+P_{3}W_{23})$$ (12)

When $P_{3}=\frac{P_{1}L_{21}+C_{2}}{R_{2}+W_{23}},\;F(P_{2}\equiv 0)$, which means that all cases are evolutionary steady states. When $P_{3}\neq \frac{P_{1}L_{21}+C_{2}}{R_{2}+W_{23}}$, let F(P_2_) = 0, which gives P_2_ = 0 and P_2_ = 1 as the two equilibrium points. Finding the derivative of F(P_2_), it follows that,

$$\frac{dF(P_{2})}{dP_{2}}=(1-2P_{2})(P_{3}R_{2}-P_{1}L_{21}-C_{2}+P_{3}W_{23})$$ (13)

When $P_{3}>\frac{P_{1}L_{21}+C_{2}}{R_{2}+W_{23}},\;P_{2}=1$ is the equilibrium point and media promote is the evolutionary stable strategy; When $P_{3}<\frac{P_{1}L_{21}+C_{2}}{R_{2}+W_{23}},\;P_{2}=0$ is the equilibrium point and media not promote is the evolutionary stable strategy.

Based on people participate

Suppose that the participate of people is N_11_, the not participate of people is N_12_, so:

$$N_{11}=P_{1}P_{2}(R_{3}-C_{3}+V_{32}+V_{31})+P_{1}(1-P_{2})(R_{3}-C_{3}+V_{31})+(1-P_{1})P_{2}(R_{3}-C_{3}+V_{32}-L_{3})+(1-P_{1})(1-P_{2})(R_{3}-C_{3}-L_{3})=P_{1}V_{31}+P_{2}V_{32}+R_{3}-C_{3}-L_{3}+P_{1}L_{3}$$ (14)

$$N_{12}=P_{1}P_{2}V_{31}+P_{1}(1-P_{2})V_{31}=P_{1}V_{31}$$ (15)

Then the replication dynamic equation for the people’s choice of participate as a strategy is,

$$F(P_{3})=\frac{dP_{3}}{dt}=P_{3}(1-P_{3})(P_{2}V_{32}+R_{3}-C_{3}-L_{3}+P_{1}L_{3})$$ (16)

When $P_{1}=\frac{-P_{2}V_{32}-R_{3}+C_{3}+L_{3}}{L_{3}},\;F(P_{3}\equiv 0)$, which means that all cases are evolutionary steady states. When , let F(P_3_) = 0, which gives P_3_ = 0 and P_3_ =1 as the two equilibrium points. Finding the derivative of F(P_3_), it follows that,

$$\frac{dF(P_{3})}{dP_{3}}=(1-2P_{3})(P_{2}V_{32}+R_{3}-C_{3}-L_{3}+P_{1}L_{3})$$ (17)

When $P_{1}>\frac{-P_{2}V_{32}-R_{3}+C_{3}+L_{3}}{L_{3}},\;P_{3}=1$ is the equilibrium point and people participate is the evolutionary stable strategy; When $P_{1}<\frac{-P_{2}V_{32}-R_{3}+C_{3}+L_{3}}{L_{3}},\;P_{3}=0$ is the equilibrium point and people not participate is the evolutionary stable strategy.

#### 4.3.3. Guidance given by the model

When the ratio of the total cost of Internet users’ participation minus the difference between the total benefit brought to people’s participation by the people individually and promoted by the online media and the loss L_3_ of people’s participation in the government but not management is greater than the probability of government management, P_1_→0, i.e., people will eventually choose not to participate in the discussion of the online opinion event. Therefore, the government can increase the cost of people’s participation C_3_ and the loss W_1_ of people’s participation in the government but not management, or decrease the gain R_1_ of people’s participation and the expected gain P_2_V_32_ of Internet users’ participation and the promotion of online media for them, to induce Internet users to reduce the probability of participating in online opinion events and thus reduce the real influence of negative online opinion events on society. Conversely, the influence of positive public opinion on offline behavior can be increased to promote tourism development and economic development in Province Y.

When the ratio of the total cost of online media promotion to the sum of online media promotion gain and online media blocking loss is greater than the probability of people’s participation, P_2_→0, i.e., the online media will choose the no-promotion strategy. Therefore, the government can, on the one hand, reduce the probability of people’s participation; on the other hand, it can increase the cost of online media promote C_2_ and increase the loss of online media promote L_21_, or reduce the gain of online media promote R_2_ and reduce the loss of online media blocking W_23_, to prompt online media to choose the blocking strategy and thus reduce the heat of negative online opinion events. If the perceived public opinion is positive, the opposite strategy can be used to promote the spread of positive public opinion, optimize the cyberspace environment, and promote regional economic development.

When the ratio of government management cost C_1_, government non-management loss L_1_, and government management gain R_1_ to online media-promoted online opinion events and government management gain L_21_ is less than the online media-promoted probability, P_1_→1, i.e., the government will choose management strategy. Therefore, reducing the cost of government management C_1_, increasing the loss of government non-management L_1_, increasing the gain of government management R_1_, and increasing the gain of government management L_21_ when the online media push will prompt the government to improve the probability of management and management level. Government management is not only reflected in the management of public opinion, but also in the management of offline businesses and the governance of cyberspace.

When an online public opinion event occurs, netizens often hope that the government will properly manage it. However, if the government fails to do so, it can lead to a heightened sense of concern and even emergency situations that threaten social stability. Therefore, measures must be taken to reduce the participation rate of individuals in discussions of negative online events. The government can increase the cost of participation in such discussions, for example, by requiring individuals to participate in compulsory forums or by increasing the cost of internet access. Improving individuals’ ability to interpret and judge information can also reduce the satisfaction and related benefits of participating in such discussions. By decreasing the participation rate, the negative impact of online public opinion events on society can be minimized, while positive public opinion can be leveraged to promote tourism and economic development in Province Y.

It is important to note that while the government can take measures to manage online public opinion, it should also respect the right of citizens to express their opinions and participate in online discussions. Therefore, any measures taken should be balanced and not infringe upon freedom of speech and expression.

Moreover, instead of just focusing on reducing the negative impact of online public opinion events, the government can also take steps to promote positive public opinion and encourage online discussions that contribute to the development and progress of society. This can be achieved through measures such as promoting civic education, providing accurate and timely information, and creating platforms for constructive dialogue between the government and the public.

Overall, effective management of online public opinion requires a comprehensive and balanced approach that takes into account the interests of all stakeholders, including the government, online media, and the public.

It is important to note that while the government’s management of online public opinion events is necessary, it should not infringe on citizens’ freedom of speech and expression. The government should strive to strike a balance between managing negative online public opinion events and respecting the rights of netizens. In addition, promoting transparency and accountability in government actions and decisions can help build trust and credibility with the public, which in turn can reduce the frequency and intensity of negative online public opinion events.

## 5. Conclusion, limitations and future work

### 5.1. Conclusion

The online public opinion perception and regulation framework proposed in this paper, which utilizes machine learning algorithms and game theory, represents a significant contribution to the field of cyberspace governance. The incorporation of LDA and neural network algorithms enhances the accuracy of detecting and perceiving public opinion topics and emotions, thereby enabling a deeper understanding of public attitudes and opinions. At the same time, the application of game theory provides effective solutions for managing public opinion and influencing public behavior, ultimately optimizing cyberspace governance, promoting regional economic development, and ensuring sustainability.

Overcoming the challenge of the cost of data collection is considered crucial in the online perception of public opinion. To overcome this obstacle, we have successfully applied the active learning strategy within machine learning. This approach not only significantly reduces the cost of data collection and human resources, but also maintains a high level of accuracy. As a result, our research presents a comprehensive and systematic solution to the problem of public opinion and online public opinion perception, achieving the goals of improving the cyberspace environment, promoting regional sustainability, and minimizing costs. This achievement has considerable economic value and practical significance.

The proposed framework has significant implications for managing online public opinion and maintaining social stability. Through an in-depth analysis of the intricate relationship between people’s will, public opinion, and behavior in the current state of cyberspace governance, we find that online public opinion management is crucial, but faces challenges in terms of perception, management, and cost.

This study conducts a comprehensive analysis of public opinion data from two representative regions in China: Province Y and Province G. The research reveals significant differences between the two provinces in terms of their economies and development. The primary concerns of the people in both regions revolve around hot topics related to the Covid-19 epidemic, including changes in the epidemic and policies for prevention and control. In addition, Province Y’s tourism industry is attracting attention, with specific destinations and tourism management being the focus of public interest. However, sentiment analysis indicates that the public has a generally negative attitude toward tourism management in Province Y, expressing dissatisfaction with its disorganized state. This dissatisfaction has the potential to negatively affect tourism development in the region, leading to a slowdown in economic growth, which hinders the pursuit of sustainable development. The proposed sentiment analysis algorithm in this paper demonstrates an accuracy of 0.925 and an F1 score of 0.924, showing a commendable model performance. Furthermore, with the introduction of an active learning strategy, the accuracy rate becomes 0.911 and the F1 score becomes 0.910, indicating the feasibility of the active learning strategy.

In conclusion, our proposed framework for online public opinion perception and regulation, which utilizes machine learning algorithms and game theory, provides valuable insights into identifying and effectively managing potential priorities through evolutionary games. In the perception part, machine learning algorithms such as LDA, CNN, and LSTM are used, and active learning is introduced to reduce the cost of perceiving public opinion. Then, an in-depth analysis of the relationship between the will of the people, Internet public opinion, and people’s behavior is performed, and based on this analysis, evolutionary games are used to model the relationship, and finally, management strategies are obtained. By analyzing public opinion data from two economically unbalanced regions in China, this study provides important suggestions for promoting sustainable and balanced regional development. Moreover, our framework can be extended to other regions to help identify relevant issues and support regional management and sustainable development. In the digital information age, the importance of public opinion continues to grow, making effective perception and management of public sentiment critical to successful regional development. Thus, our comprehensive study on the perception and management of online public opinion has significant academic significance and practical applications.

### 5.2. Limitations

The analysis of search data reveals a certain degree of randomness, particularly in the context of covid-19 information, which has the potential to exert a significant influence on the prevailing opinion on the internet. Furthermore, the lack of access to years of data for analysis represents a limitation in this field of study. The three-party game model proposed in this paper is not sufficiently comprehensive to account for the factors influencing the behavior of the three participating subjects in the evolution of online public opinion. Consequently, it is not yet possible to provide a complete explanation or reduction of the evolution of internet public opinion. Furthermore, the framework proposed in this paper should be employed with caution, given the sensitivities that influence public opinion. It is important to note that the objective of this paper is to validate the methodology and not to assess the liability or associated risks of the framework.

### 5.3. Future work

In future research, we suggest exploring the following directions:

Investigate management methods: Building on the game theory framework, there is room for further research on specific management methods for online public opinion. In particular, we suggest exploring the application of blockchain technology, which can leverage its distributed thinking and trust mechanism. The inherent decentralized nature and tamper-proof features of blockchain can significantly enhance the dissemination and credibility of public opinion information, leading to more effective network public opinion management. At the same time, the appropriate use of frames should be explored to avoid inappropriate influences on public opinion.

The objective of this study is to optimize the active learning strategy. Although the existing active learning strategy has the effect of reducing the cost of data collection, there is nevertheless scope for further improvement. It is recommended that the integration of more efficient sample selection algorithms be considered as a means of improving the accuracy and efficiency of active learning while simultaneously reducing the burden of data labeling.

The objective of fostering interdisciplinary collaboration is to facilitate the integration of diverse perspectives and approaches from different academic disciplines. By integrating the framework of online public opinion perception and regulation with other disciplinary fields, such as sociology, psychology, and communication science, we can gain a more profound comprehension of the fundamental causes and the intricate mechanisms that underpin the formation of online public opinion. This cross-disciplinary approach will facilitate the acquisition of more profound insights and the development of novel solutions for the management of online public opinion.
